# Matrix metalloproteinase 13 modulates intestinal epithelial barrier integrity in inflammatory diseases by activating TNF

**DOI:** 10.1002/emmm.201202100

**Published:** 2013-05-30

**Authors:** Roosmarijn E Vandenbroucke, Eline Dejonckheere, Filip Van Hauwermeiren, Sofie Lodens, Riet De Rycke, Elien Van Wonterghem, An Staes, Kris Gevaert, Carlos López-Otin, Claude Libert

**Affiliations:** 1 Department for Molecular Biomedical Research, VIBGhent, Belgium; 2 Department of Biomedical Molecular Biology, Ghent UniversityGhent, Belgium; 3 Department of Medical Protein Research, VIBGhent, Belgium; 4 Department of Biochemistry, Ghent UniversityGhent, Belgium; 5 Departamento de Bioquimica y Biologia Molecular, Instituto Universitario de Oncologia, Universidad de OviedoOviedo, Spain

**Keywords:** IBD, intestinal permeability, matrix metalloproteinase, sepsis, tumour necrosis factor

## Abstract

Several pathological processes, such as sepsis and inflammatory bowel disease (IBD), are associated with impairment of intestinal epithelial barrier. Here, we investigated the role of matrix metalloproteinase MMP13 in these diseases. We observed that MMP13^−/−^ mice display a strong protection in LPS- and caecal ligation and puncture-induced sepsis. We could attribute this protection to reduced LPS-induced goblet cell depletion, endoplasmic reticulum stress, permeability and tight junction destabilization in the gut of MMP13^−/−^ mice compared to MMP13^+/+^ mice. Both *in vitro* and *in vivo*, we found that MMP13 is able to cleave pro-TNF into bioactive TNF. By LC-MS/MS, we identified three MMP13 cleavage sites, which proves that MMP13 is an alternative TNF sheddase next to the TNF converting enzyme TACE. Similarly, we found that the same mechanism was responsible for the observed protection of the MMP13^−/−^ mice in a mouse model of DSS-induced colitis. We identified MMP13 as an important mediator in sepsis and IBD via the shedding of TNF. Hence, we propose MMP13 as a novel drug target for diseases in which damage to the gut is essential.

→ See accompanying article http://dx.doi.org/10.1002/emmm.201302899

## INTRODUCTION

Matrix metalloproteinases (MMPs) are important mediators during the process of inflammation and are consequently involved in several pathological processes, such as cancer (Decock et al, 2011; Overall & Lopez-Otin, 2002), sepsis (Vandenbroucke et al, [Bibr b73],[Bibr b75]; Vanlaere & Libert, 2009), lung diseases (Vandenbroucke et al, 2011b), ischaemia/reperfusion (Dejonckheere et al, 2011) and arthritis (Burrage et al, 2006). They constitute a group of structurally and functionally related zinc-dependent endopeptidases responsible for cleaving and rebuilding connective tissue components such as collagen, elastin, gelatin and casein (Zitka et al, 2010). Moreover, several extracellular and intracellular non-matrix substrates of MMPs have been identified, including chemokines, cytokines, growth factors, junction proteins, molecular chaperones and cytoskeletal proteins (Cauwe & Opdenakker, 2010; Cauwe et al, 2007).

MMP13 (collagenase-3) belongs to the family of collagenases together with MMP1 and MMP8. Matrix substrates of MMP13 include native collagen, gelatin and aggrecan and non-matrix substrates include MCP-3 and pro-MMP9. MMP13 activity in chondrocytes and synovial cells appears to be critical in cartilage formation and in joint diseases (Takaishi et al, 2008). MMP13 has also been implicated in tumour invasion and metastasis (Fukuda et al, 2011; Wu et al, 2012), lung diseases (Shukla et al, 2006), and periodontal disease (de Aquino et al, 2009). Additionally, some reports suggest that MMP13 is important in IBD, an umbrella term that includes Crohn's disease and ulcerative colitis (UC), two chronic relapsing inflammatory disorders of the gut. Significantly increased levels of MMP13 mRNA were found in IBD biopsy specimens (Rath et al, 2006). Moreover, a positive correlation was found between MMP13 expression and the histological inflammation scores in mucosal samples from IBD patients (Vizoso et al, 2006). Recently, both endothelial cells and infiltrating leucocytes were identified as the major sources of MMP13 in UC (Rath et al, 2010).

IBD, as well as other pathological events such as sepsis, are associated with impairment of the intestinal epithelial barrier (John et al, 2011; Turner, 2009). Consequently, intestinal lumen components (*i.e*., bacteria, PAMPs and alarmins) leak into the bloodstream, inducing a systemic inflammatory response syndrome (SIRS), which can result in lethal multi-organ failure (Balzan et al, 2007). Tight junctions play an important role in the formation and maintenance of the intestinal epithelial barrier, as they tightly seal adjacent intestinal epithelial cells (IECs) at the apical site (Farquhar & Palade, 1963). Tight junctions transmembrane proteins such as occludin and claudins mediate adhesion by linkage to the underlying plaque proteins, which in turn associate with the cytoskeleton. Examples of plaque proteins are zona occludens (ZO) 1, −2 and −3 (Niessen, 2007). Several papers have reported that MMPs play a direct or indirect role in increasing epithelial barrier permeability (Ailenberg & Sefton, 2009; Huet et al, 2011; Vandenbroucke et al, 2012).

In this study, we investigated whether MMP13 plays a role in epithelial barrier disruption during pathological events such as sepsis and IBD, by using endotoxemia and DSS-induced colitis models, respectively. We found that MMP13 contributes to intestinal permeability by causing TNF shedding, which increases the levels of soluble, bioactive TNF. Consequently, TNF induces endoplasmic reticulum (ER) stress mediated mucus depletion in the gut, increased intestinal inflammation, reduced functionality of the tight junctions, and increased intestinal permeability. These changes lead to leakage of luminal components into the periphery, systemic inflammation, organ damage and eventually death. In conclusion, our results suggest that MMP13 is a potential therapeutic target for treatment of inflammatory disorders associated with TNF-dependent dysfunction of the intestinal barrier, such as sepsis and IBD.

## RESULTS

### MMP13 deficiency protects mice from LPS-induced systemic inflammation and lethality

**Figure 1:**
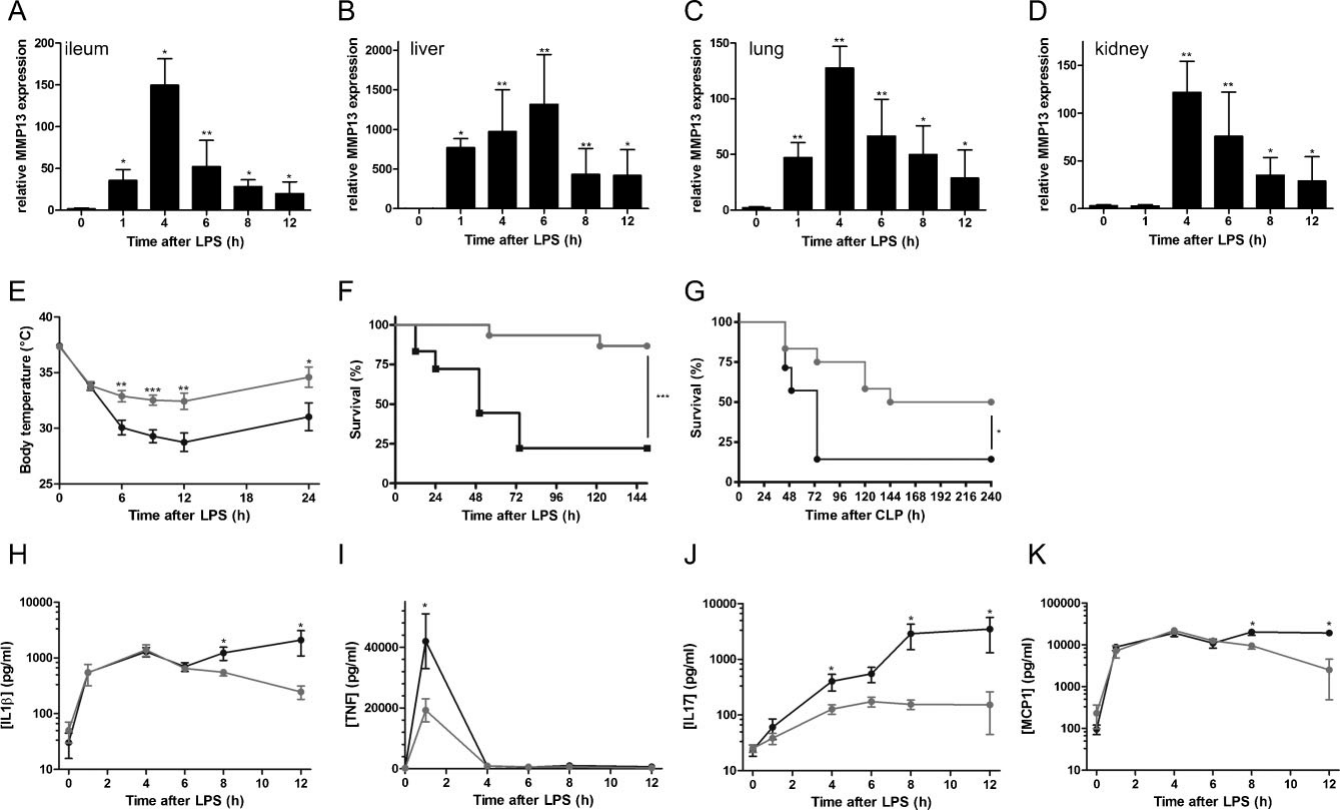
MMP13 deficiency protects mice from LPS-induced systemic inflammation and lethality **A–D.** MMP13 gene expression analysis in ileum (A), liver (B) lung (C), and kidney (D) 0, 1, 4, 6, 8, and 12 h after LPS challenge (*n* = 5).**E–F.** Body temperature (E) and survival (F) of MMP13^+/+^ (black) and MMP13^−/−^ (grey) mice after LPS injection (*i.p*.; 17.5 mg/kg) (*n* = 15).**G.** Survival of MMP13^+/+^ (black; *n* = 7) and MMP13^−/−^ (grey; *n* = 12) mice after CLP.**H–K.** Serum cytokine and chemokine levels after injection of LPS in MMP13^+/+^ (black) and MMP13^−/−^ (grey) mice: IL1β (H), TNF (I), IL17 (J) and MCP1 (K) (*n* = 4–5).

To investigate whether MMP13 contributes to the lethal effects of sepsis, we compared the response of wild type (MMP13^+/+^) and MMP13-deficient (MMP13^−/−^) mice in the endotoxemia model, *i.e*., i.p. injection of LPS, a model of human sepsis (Cantaluppi et al, 2008; Cruz et al, [Bibr b12],[Bibr b13]). Early after endotoxemia induction, we observed strong up-regulation of MMP13 in all tested organs (Fig 1A–D). MMP13^−/−^ mice were significantly protected against LPS-induced hypothermia (Fig 1E) and death (Fig 1F). We also subjected MMP13^+/+^ and MMP13^−/−^ mice to caecal ligation and puncture (CLP), the gold standard model for human sepsis (Dejager et al, 2011), and this revealed that MMP13^−/−^ mice are also significantly protected against CLP-induced death (Fig 1G). Next, we studied the cyto- and chemokine levels in sera of LPS-injected mice. We observed substantial differences in the absolute concentrations, time courses and clearance of the tested cyto- and chemokines. Most inflammatory mediators reached their maximum around 6–12 h, with the exception of TNF, which reached a peak after 1 h, followed by a rapid decline (Fig 1H–K and Supporting Information Fig S1). We observed that 12 h after LPS injection, all cytokines (IL1β, IL6, IL10, IL12p40, IL12p70, IL17 and IFN-γ) and chemokines (G-CSF, GM-CSF, KC, MCP1 and Rantes) were lower in MMP13^−/−^ mice than in MMP13^+/+^ mice. Moreover, serum levels of IL1β (Fig 1G), TNF (Fig 1H), IL17 (Fig 1I) and MCP1 (Fig 1J) were lower even at the earlier time points after challenge in MMP13^−/−^ mice. These results indicate that MMP13 plays a detrimental role in sepsis-induced systemic inflammation and lethality.

### MMP13 deficiency protects mice from LPS-induced intestinal permeability, mucus depletion and ER stress

The ‘gut as motor of sepsis’ hypothesis has been postulated based on the importance of the intestinal epithelium as a physical barrier between the intestinal lumen and the immune cells in the lamina propria (Carrico et al, 1986; Deitch & Berg, 1987). Indeed, in critically ill sepsis patients, intestinal permeability is correlated with bacterial translocation and subsequent multi-organ failure (Faries et al, 1998; Swank & Deitch, 1996).

**Figure 2:**
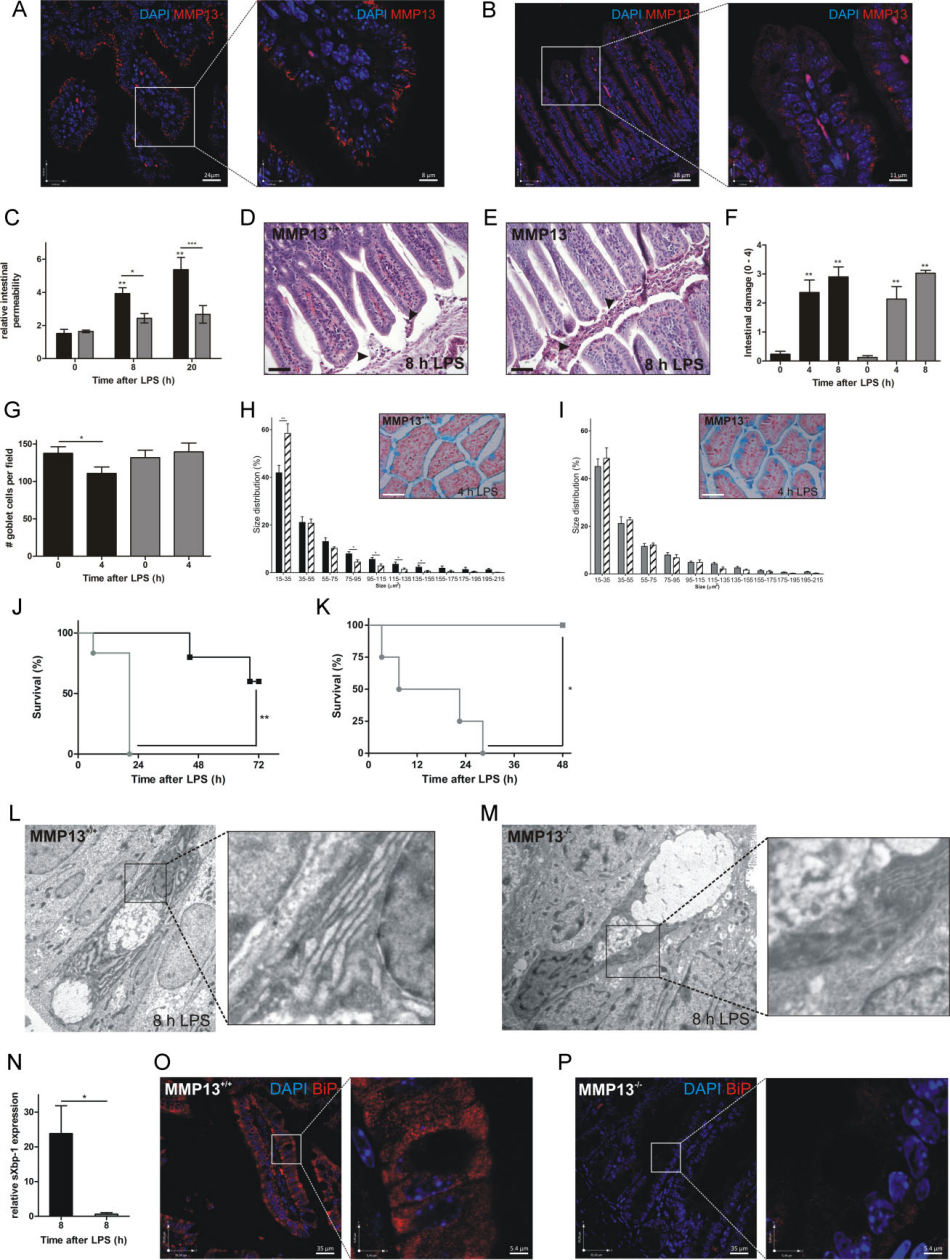
MMP13 deficiency protects mice from LPS-induced intestinal permeability, mucus depletion and ER stress **A,B.** Representative confocal images of MMP13 (red) and DAPI (blue) immunostaining of unstimulated (A) and LPS-stimulated (B) (4 h) ileum sections.**C.** Intestinal permeability in MMP13^+/+^ (black) and MMP13^−/−^ (grey) mice 0, 8 and 20 h after LPS injection. Fluorescently labeled FITC-dextran was administered orally to the mice and 5 h later plasma was collected and fluorescence was measured.**D,E.** Representative hematoxylin and eosin stained sections of the ileum of MMP13^+/+^ (D) and MMP13^−/−^ (E) 8 h after LPS injection. Bar = 50 µm. The presence of epithelial cells and cell debris in the lumen is indicated with an arrow.**F.** Quantification of intestinal tissue damage in MMP13^+/+^ (black) and MMP13^−/−^ (grey) mice: four neutral observers evaluated ileal sections stained with hematoxylin and eosin (*n* = 5) in a blinded setup. LPS-induced intestinal damage is characterized by decreased villus height, disappearance of the mucus layer and goblet cells along the villus, cell death at the villus top, and cell debris in the lumen.**G–I** Goblet cell amount (G) and size distribution (H-I) in MMP13^+/+^ (black; H) and MMP13^−/−^ (grey; I) mice 0 (blank) and 4 h (hatched) after LPS injection. The insert displays a representative image of Alcian blue stained ileal sections of MMP13^+/+^ (H) and MMP13^−/−^ (I) mice. Bar = 50 µm.**J.** Survival of MMP13^+/+^ mice (black) and MMP13^+/+^ mice pretreated with the mucus depleting agent pilocarpine (grey) after LPS injection (i.p.; 6 mg/kg; LD50 in MMP13^+/+^) (*n* = 5).**K.** Survival of LPS-injected (i.p.; 17.5 mg/kg; LD0 in MMP13^−/−^ mice) untreated (square; *n* = 4) and pilocarpine treated (circle; *n* = 3) MMP13^−/−^ mice (grey).**L,M.** Representative morphological TEM images of MMP13^+/+^ (L) and MMP13^−/−^ (M) mice 8 h after LPS injection. The insert is a close-up of the ER.**N.** mRNA expression analysis of spliced Xbp-1 (sXbp-1) in ileum lysates of MMP13^+/+^ (black) and MMP13^−/−^ (grey) 8 h after LPS injection (*n* = 5).**O,P.** Representative confocal images of BiP (red) and DAPI (blue) immunostaining of MMP13^+/+^ (O) and MMP13^−/−^ (P) ileum sections 8 h after LPS injection.

We first addressed which cell type in the ileum is responsible for MMP13 expression. Immunostaining revealed MMP13 expression both in epithelial and inflammatory cells (Fig 2A). Four hours of LPS stimulation resulted in decreased MMP13 staining in the epithelial cells at the top of the villi, which suggests LPS-induced MMP13 secretion, while MMP13 expression in the inflammatory cells in the lamina propria increased further after LPS injection (Fig 2B).

To exclude compensation by other MMPs in the ileum of MMP13^−/−^ mice, we analysed MMP expression in ileum lysates. None of the tested MMPs were up-regulated, except MMP3 which displayed a two-fold down-regulation in MMP13^−/−^ lysates when compared to MMP13^+/+^ mice (Supporting Information Fig S2).

To study intestinal leakage in MMP13^+/+^ and MMP13^−/−^ mice after LPS challenge, fluorescently labeled FITC-dextran was administered orally to the mice. We observed an increase of fluorescence in plasma of LPS-treated MMP13^+/+^ mice, which is a measure of leakage from the intestinal lumen into the peripheral blood (Fig 2C). In contrast, MMP13^−/−^ mice did not suffer from increased intestinal permeability. Despite the difference in intestinal permeability, morphological analysis revealed similar intestinal damage in MMP13^+/+^ and MMP13^−/−^ mice after LPS challenge (Fig 2D–F). Similarly, TUNEL staining showed equal levels of apoptosis induction in MMP13^+/+^ and MMP13^−/−^ mice (Supporting Information Fig S3). However, detailed analysis of the goblet cells by Alcian blue and MUC2 staining did reveal important differences both in the amount and size of Alcian blue positive goblet cells early after LPS challenge. Fig 2G shows that LPS reduces the amount of Alcian blue positive goblet cells in MMP13^+/+^ mice, but not in MMP13^−/−^ mice. Additionally, size distribution analysis shows that the percentage of goblet cells with reduced mucus content increases substantially in MMP13^+/+^ mice 4 h after LPS challenge, whereas size distribution was unaffected in MMP13^−/−^ mice (Fig 2H–I). Those observations were further confirmed by MUC2 immunostaining, both at 4 and 24 h (Supporting Information Fig S4). Treatment with pilocarpine, a mucus depleting agent (Albanese et al, 1994), sensitized both MMP13^+/+^ (Fig 2J) and MMP13^−/−^ (Fig 2K) mice to endotoxemia, pointing towards a crucial role of mucus in the protection against sepsis. More detailed analysis of goblet cell morphology by transmission electron microscopy (TEM) revealed that the ER of wild type goblet cells was dilated after LPS challenge (Fig 2L). This was not the case in MMP13^−/−^ mice (Fig 2M). Additionally, two markers for ER stress, namely spliced Xbp1 (sXbp1) mRNA (Fig 2N) and BiP protein (Fig 2O–P) were higher in LPS-stimulated MMP13^+/+^ compared to MMP13^−/−^ mice. These results show that MMP13 deficiency protects from LPS-induced intestinal permeability, mucus depletion and goblet cell ER stress after systemic LPS injection.

### MMP13 contributes to tight junction destabilization

**Figure 3:**
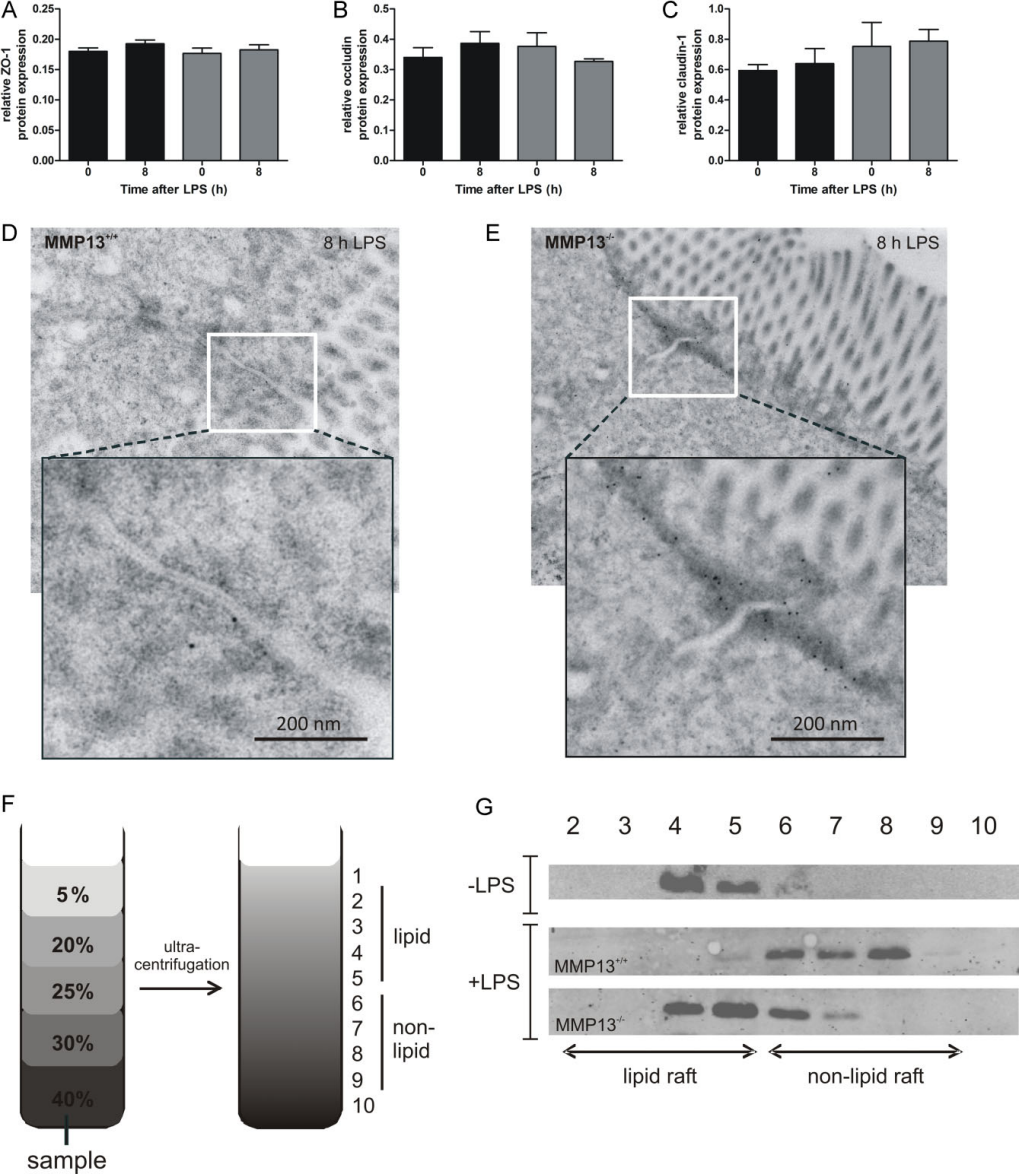
MMP13 contributes to tight junction destabilization Source data is available for this figure in the Supporting Information. **A–C.** Western blot quantification of the protein expression levels of ZO-1 (A), occludin (B) and claudin (C) in mucosal scrapings of ilea from MMP13^+/+^ (black) and MMP13^−/−^ (grey) mice 0 and 8 h after LPS injection (*n* = 3–4).**D,E.** Representative ZO-1 immunogold-labeled TEM images of MMP13^+/+^ (D) and MMP13^−/−^ (E) mice 8 h after LPS injection.**F.** Schematic overview of the separation of the lipid raft and non-lipid-raft fractions by ultracentrifugation.**G.** Caveolin-1 western blot analysis of fractions obtained after ultracentrifugation of mucosal scrapings from MMP13^+/+^ and MMP13^−/−^ mice 0 and 8 h after LPS challenge.

Intercellular junctions, such as tight junctions, play a crucial role in the formation and maintenance of the intestinal epithelial barrier. Consequently, intestinal permeability is often linked to tight junction malfunctioning (John et al, 2011). Several MMPs have been shown to cleave the components of tight junctions, thereby influencing their functionality. For example, it has been reported that hypoxia-induced MMP13 results in disorganization and fragmentation of the intracellular tight junction scaffolding protein ZO-1 (zona occludens), which results in hyperpermeablility of the blood–brain barrier (Lu et al, 2009). Western blot analysis of intestinal mucosal scrapings showed that LPS did not induce a decrease in the amount of full length ZO-1 protein (Fig 3A). Similar results were obtained for occludin (Fig 3B) and claudin-1 (Fig 3C). However, IEM analysis did reveal severe differences in the amount of immunogold-labeled ZO-1 located at the apical tight junctions: after LPS challenge, much more tight junction-localized ZO-1 could be detected in the IECs of MMP13^−/−^ mice compared to MMP13^+/+^ mice (Fig 3D–E). This observation was confirmed by immunofluorescent ZO-1 staining (Supporting Information Fig S5). Apparently, although total ZO-1 protein levels were equal in MMP13^+/+^ and MMP13^−/−^ mice, there was a profound difference in localization after LPS challenge.

Tight junctions are part of specialized lipid raft-like membrane microdomains (Nusrat et al, 2000). It was recently shown that sepsis causes redistribution of tight junctions in membrane microdomains and that this redistribution is responsible for increased permeability (Li et al, 2009). Mucosal scrapings from the ileum were homogenized and separated by ultracentrifugation. As shown in Fig 3F, after ultracentrifugation, the upper fractions contain the lipid rafts and the lower fractions the non-lipid rafts. Comparison of mucosal scrapings from the ilea of challenged MMP13^+/+^ and MMP13^−/−^ mice revealed that caveolin-1, an essential molecule in lipid raft microdomains, was present in different fractions. In LPS-treated MMP13^−/−^ mice, caveolin-1 was in the lipid raft fraction, but it shifted almost completely to the non-lipid-raft fraction in the LPS-treated MMP13^+/+^ mice (Fig 3G). This indicates that MMP13 deficiency protects from LPS-induced tight junction redistribution and therefrom resulting increased intestinal permeability.

### MMP13 cleaves proTNF, resulting in the formation of mature, bioactive TNF

Marchiando et al. recently showed that TNF induces caveolin-1-dependent endocytosis of tight junction proteins, which results in increased intestinal permeability (Marchiando et al, 2010). TNF is translated as a 26-kDa transmembrane precursor protein (tmTNF) that is proteolytically cleaved to release the soluble and biologically active 17-kDa C-terminal part (soluble TNF; sTNF). sTNF is known to induce a concentration- and time-dependent increase in epithelial permeability both *in vitro* and *in vivo* (Gitter et al, 2000; He et al, 2012; Ma et al, [Bibr b46],[Bibr b47]; Marchiando et al, 2010; Schmitz et al, 1999). Although the major sheddase responsible for proTNF cleavage is TACE (tumour necrosis factor-α-converting enzyme; Adam17), Adam10 (Le Gall et al, 2009) and MMP-mediated shedding of TNF might also be important (Overall & Blobel, 2007). Several MMPs were shown to be able to cleave proTNF *in vitro*: MMP1, −2, −3, −7, −9, −12, −14 and −15 (Chandler et al, 1996; d'Ortho et al, 1997; Gearing et al, 1995; Tam et al, 2004).

**Figure 4:**
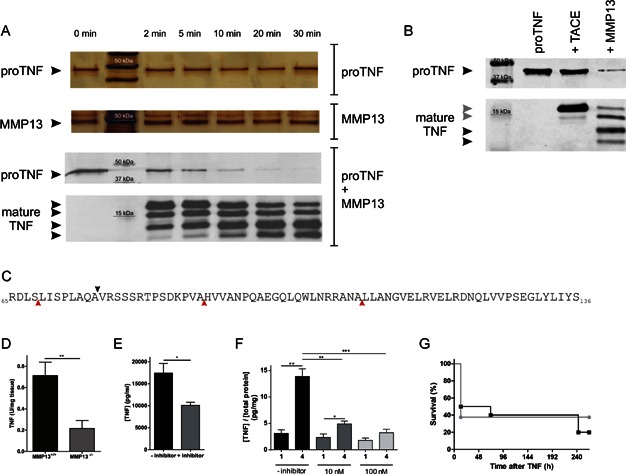
MMP13 cleaves proTNF to generate mature, bioactive TNF Source data is available for this figure in the Supporting Information. Silver staining of proTNF (47 kDa) and MMP13 (52 kDa) samples incubated at 37°C for 0, 2, 5, 10, 20 and 30 min followed by SDS-PAGE. Anti-TNF western blot analysis of co-incubated proTNF (47 kDa) and MMP13 (52 kDa) samples at 37°C for 0, 2, 5, 10, 20 and 30 min with formation of mature TNF.Anti-TNF western blot analysis of proTNF (47 kDa), incubated at 37°C for 10 min with TACE and MMP13. Cleavage of proTNF by TACE results in two mature TNF fragments (∼17 and ∼15 kDa; grey). Cleavage of proTNF by MMP13 results in four cleavage fragments (∼17, ∼15, ∼13 and ∼11 kDa).Identified proTNF cleavage sites of MMP13 by LC-MS/MS (red arrow) and known TACE cleavage site (black arrow).TNF bioactivity of ileal lysates of MMP13^+/+^ (black) and MMP13^−/−^ (grey) mice 1 h after LPS injection (*n* = 5).TNF levels of *in vitro* LPS-stimulated MMP13^+/+^ macrophages incubated in the absence or presence of MMP13 inhibitor.TNF levels of ileum explants from *in vivo* LPS stimulated MMP13^+/+^ mice incubated *ex vivo* with and without MMP13 inhibitor.Survival of MMP13^+/+^ (black) and MMP13^−/−^ (grey) mice injected with TNF (25 µg/20 g) (*n* = 8).

To study whether MMP13 can cleave proTNF, the two proteins were co-incubated *in vitro*. As shown in Fig 4A and B, MMP13 can cleave proTNF *in vitro* to generate different N-terminal TNF fragments (∼17, ∼15, ∼13 and ∼11 kDa). Comparison of the TNF bioactivity revealed that 10 min co-incubation of proTNF with TACE or activated MMP13 (1:1 ratio) results in 218 and 227 U/mg bioactive TNF, respectively. By LC-MS/MS analysis, we could identify three different MMP13 cleavage sites, namely S68, A90 and A111 (Fig 4C). To determine if shedding of TNF is also dependent on MMP13 *in vivo*, we analysed TNF bioactivity in ileal lysates from MMP13^+/+^ and MMP13^−/−^ mice obtained 1 h after LPS challenge. Fig 4D shows that TNF bioactivity was significantly higher in ileum of MMP13^+/+^ mice, which suggests that MMP13 can also cleave proTNF into soluble, bioactive TNF *in vivo*.

MMP13-dependent TNF cleavage was further confirmed both *in vitro* and *ex vivo*. Primary mouse macrophages from MMP13^+/+^ mice were isolated by peritoneal lavage and stimulated *in vitro* with LPS in the absence or presence of MMP13 inhibitor. As shown in Fig 4E, MMP13 inhibition results in a decrease in TNF release into the supernatant. Similarly, ileum explants of LPS injected MMP13^+/+^ mice were incubated *ex vivo* with different concentrations of MMP13 inhibitor. This resulted in a dose dependent decrease in TNF (Fig 4F).

If the LPS resistance of the MMP13^−/−^ mice can be attributed to TNF, one would expect that MMP13^−/−^ mice are as sensitive to systemic TNF injection as MMP13^+/+^ mice. Indeed, no significant difference in response of MMP13^+/+^ and MMP13^−/−^ mice upon TNF injection could be detected (Fig 4G).

### MMP13-dependent intestinal TNF activation results in mucus depletion and increased intestinal inflammation

**Figure 5:**
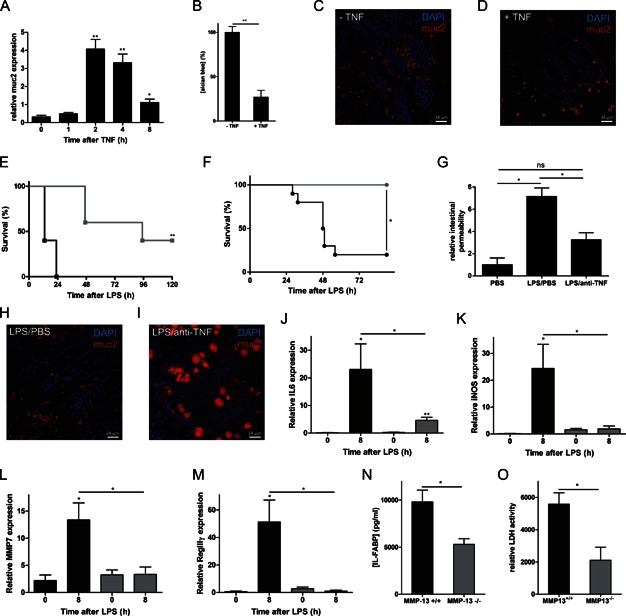
MMP13-dependent intestinal TNF activation results in mucus depletion, increased intestinal inflammation, systemic inflammation and organ damage **A.** TNF (2000 U/ml) induces upregulation of Muc2 gene expression in goblet cells *in vitro* (*n* = 6).**B.** Quantification of total goblet cell mucus before and 8 h after TNF injection *in vivo* by Alcian blue staining of isolated ileum samples, followed by laxative treatment and absorbance measurement of the supernatant (*n* = 8).**C,D.** Representative images of MUC2 (red) and DAPI (blue) immunostainings of ileal sections in the absence of TNF (C) and 8 h after TNF injection (D).**E.** Survival curve of non-sterile (black) and sterile, antibiotics treated (grey) mice after LPS injection (*i.v*.; 250 µg/20 g) (*n* = 12).**F,G.** Survival curve (F) and intestinal permeability (G) of LPS-injected MMP13^+/+^ mice pretreated with PBS (black; *n* = 10) or anti-TNF (grey; *n* = 3) (*i.p*.; 17.5 mg/kg).**H,I.** Representative mucin-2 (red) confocal images of ileal sections of LPS-injected mice pretreated with PBS (H) and anti-TNF (I).**J,K.** Gene expression analysis of IL6 and iNOS in ileum lysates of MMP13^+/+^ (black) and MMP13^−/−^ (grey) mice 0 and 8 h after LPS injection (*n* = 4–5).**L,M.** Gene expression analysis of the Paneth-cell-specific genes MMP7 (H) and RegIIIγ (I) in ileum lysates of MMP13^+/+^ (black) and MMP13^−/−^ (grey) mice 0 and 8 h after LPS injection (*n* = 4–5).**N.** IL-FABP levels in serum of MMP13^+/+^ (black) and MMP13^−/−^ (grey) mice 8 h after LPS injection (*n* = 5–7).**O.** Relative serum LDH activity 8 h after LPS injection in MMP13^+/+^ (black) and MMP13^−/−^ (grey) mice (*n* = 6–14).

TNF has been shown to induce mucus secretion (McElroy et al, 2011), so the depletion of mucus observed in MMP13^+/+^ mice might be a direct effect of increased TNF levels. Indeed, incubation with TNF *in vitro* resulted in goblet cell Muc2 gene expression up-regulation (Fig 5A) and TNF injection *in vivo* resulted in a reduction of total goblet cell mucus (Fig 5B). The latter was confirmed by immunofluorescent staining of intestinal MUC2 before (Fig 5C) and after (Fig 5D) TNF injection. In the absence of a proper mucus layer, intestinal bacteria can interact with the underlying IECs (McGuckin et al, 2011). To address the importance of intestinal bacteria in LPS-induced shock, we first treated wild type mice with antibiotics to remove all bacteria from the gut. Next, we injected sterile and non-sterile MMP13^+/+^ mice with LPS and analysed the effect on LPS lethality and IL-6 levels. Fig 5E shows that sterile mice were protected against the lethal effects of LPS. To address the importance of TNF in the endotoxemia model, we treated mice with Etanercept that acts as a TNF inhibitor (Kerensky et al, 2012). TNF inhibition resulted in protection of the mice in terms of survival rate (Fig 5F), intestinal permeability (Fig 5G) and amount of mucus containing goblet cells (Fig 5H and I). Additionally, mice deficient in the TNF receptor TNFR1, which is implicated in TNF-induced acute toxicity (Van Hauwermeiren et al, 2011), were protected against LPS-induced lethality (Supporting Information Fig S6).

The absence of mucus can lead to increased interaction of bacteria with the IECs (McGuckin et al, 2011). This, together with elevated TNF levels, could induce epithelial cell inflammation. Indeed, we observed more intestinal inflammation in MMP13^+/+^ mice than in MMP13^−/−^ mice. LPS injection resulted in strong up-regulation of IL-6 (Fig 5J) and iNOS (Fig 5K) in the ileum of MMP13^+/+^ mice. This up-regulation was significantly lower in MMP13^−/−^ mice. Moreover, we observed increased Paneth cell activation in MMP13^+/+^ mice. This was reflected in increased MMP7 (Fig 5L) and RegIIIγ (Fig 5M) gene expression levels after LPS challenge in MMP13^+/+^ mice, while no induction of Paneth-specific genes was observed in MMP13^−/−^ mice.

### MMP13-dependent intestinal leakage results in systemic inflammation and multi-organ failure

After LPS challenge, MMP13^+/+^ mice, in contrast to MMP13^−/−^ mice, displayed an increase in intestinal permeability (Fig 2C). This increased permeability can result in leakage of intestinal lumen components into the periphery. Indeed, serum analysis of LPS-injected mice showed high ileal fatty acid-binding protein (IL-FABP) levels in MMP13^+/+^ mice (Fig 5N), a non-invasive marker for intestinal mucosal damage (Lieberman et al, 1997). IL-FABP levels were significantly lower in MMP13^−/−^ mice. Leakage of intestinal content into the periphery can induce a further increase in systemic inflammation, which eventually results in multi-organ failure. Serum levels of lactate dehydrogenase (LDH), a marker for general tissue damage, were below detection limit in unchallenged mice (data not shown). However, LDH levels were higher in MMP13^+/+^ than in MMP13^−/−^ mice 8 h after LPS challenge (Fig 5O), which suggests multi-organ failure in MMP13^+/+^, but not in MMP13^−/−^ mice.

### MMP13 deficiency results in reduced signs of clinical colitis after DSS treatment

**Figure 6:**
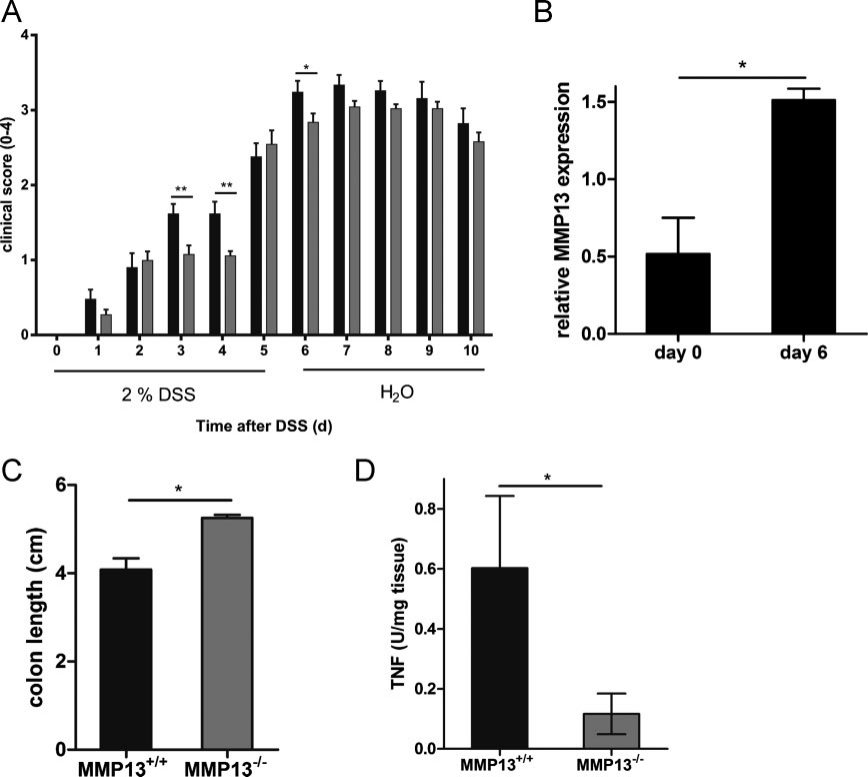
MMP13 deficiency results in reduced clinical signs of DSS-induced colitis Clinical score of MMP13^+/+^ (black) and MMP13^−/−^ (grey) mice after 5 days of treatment with 2% DSS (*n* = 12–14). Clinical scores are a composite of body weight loss, rectal bleeding and stool consistency scores.MMP13 gene expression before and 6 days after 2% DSS treatment.Colon length of MMP13^+/+^ (black) and MMP13^−/−^ (grey) mice on day 6 after the start of the DSS treatment (*n* = 4).TNF bioactivity of colon lysates of MMP13^+/+^ (black) and MMP13^−/−^ (grey) mice 6 days after 2% DSS treatment (*n* = 4; one-tailed *t*-test).

Both TNF and MUC2 play a major role in the development of DSS-induced colitis via their involvement in epithelial barrier function, and they are altered before epithelial cell damage occurs (Dharmani et al, 2011). On the basis of our findings, we hypothesized that the absence of MMP13 could be protective in this colitis model. When MMP13^+/+^ and MMP13^−/−^ mice were given 2% DSS in the drinking water for 5 days, the knockout mice showed milder disease in terms of weight loss, stool consistency and rectal bleeding (Fig 6A). In agreement with this, we observed a significant increase in MMP13 gene expression after DSS treatment (Fig 6B). The DSS-induced model of colitis is associated with a significant decrease in colon length, and evaluation of colon length is believed to be the least variable parameter. Measurement of colon length in MMP13^+/+^ and MMP13^−/−^ mice 6 days after the start of DSS treatment revealed that MMP13 deficiency reduces the effect of DSS-induced colitis on the length of the colon (Fig 6C). Again, this was associated with reduced TNF bioactivity in colon lysate (Fig 6D).

## DISCUSSION

The intestinal epithelium separates the intestinal lumen from the underlying lamina propria and thereby provides an important physical barrier against entry of luminal microbiota. Apical tight junctions are multifunctional structures that form a seal between adjacent epithelial cells and prevent paracellular diffusion of luminal material across the epithelium (Farquhar & Palade, 1963). Consequently, inflammation-induced disruption of tight junctions can contribute to leakage of luminal components into the periphery (John et al, 2011). Intestinal epithelial dysfunction is believed to be a common final pathway contributing to organ dysfunction and mortality in sepsis (Fink, 2003). In line with this, we observed a huge increase in intestinal permeability and IL-FABP serum levels, a marker for intestinal damage, in MMP13^+/+^ mice after LPS challenge. This was associated with high serum levels of cytokines, chemokines and LDH, the latter being an indicator of general tissue damage. In contrast, LPS-injected MMP13^−/−^ mice maintained a functional epithelial barrier and exhibited less severe systemic inflammation and organ damage. Eventually, this results in survival of the MMP13^−/−^ mice, while almost all MMP13^+/+^ mice succumbed. Similar protection was observed in the mouse model that is considered as the ‘gold standard’ for sepsis research, namely CLP (Dejager et al, 2011).

Both *in vivo* and *in vitro* studies have demonstrated that TNF is one of the crucial factors involved in the pathogenesis of intestinal permeability and that tight junction destabilization is implicated in this process (Gitter et al, 2000; He et al, 2012; Ma et al, [Bibr b46],[Bibr b47]; Marchiando et al, 2010; Schmitz et al, 1999). ProTNF is a transmembrane protein that is cleaved into soluble, biological active TNF. Although TACE is the main sheddase involved in this process, it is likely that TACE and MMPs have complementary roles in the rapid shedding and activation of proTNF in response to different stimuli (Overall & Blobel, 2007). This idea is strengthened by the fact that TACE^−/−^ cells still secrete 10–20% sTNF compared to TACE^+/+^ cells (Killar et al, 1999). Noncleavable transmembrane TNF transgenic mice were fully protected from endotoxic shock, pointing towards the importance of TNF cleavage in the endotoxemia model (Mueller et al, 1999). Here, we clearly show that MMP13 can cleave and activate proTNF *in vitro* and we could identify three cleavage sites, one upstream and two downstream of the TACE cleavage site, resulting in similar bioactivity. However, substrate specificity of MMPs is partly regulated by their cellular localization, which makes it dangerous to directly interpret substrates identified *in vitro* as *in vivo* substrates. We detected MMP13 expression in epithelial and inflammatory cells in the ileum and confirmed via several approaches that TNF is a direct substrate of MMP13. *In vivo*, bioactive, soluble TNF levels were higher in the inflamed tissue of MMP13^+/+^ compared to MMP13^−/−^ mice in LPS-induced shock. Moreover, LPS stimulation of both primary mouse macrophages and ileum explants incubated with MMP13 inhibitor resulted in reduced TNF levels. Additionally, MMP13^+/+^ and MMP13^−/−^ mice showed a similar response upon systemic TNF injection. The latter indicates that the resistance of the MMP13^−/−^ mice to LPS challenge can at least partially be attributed to the MMP13-dependent cleavage of TNF. To exclude the involvement of other proteases that have been described to cleave and activate TNF, we measured Adam17 and -10 as well as MMP1, −2, −3, −7, −9, −12, −14 and −15 expression (Chandler et al, 1996; d'Ortho et al, 1997; Gearing et al, 1995; Tam et al, 2004). Only MMP7 was significant higher expressed in LPS-stimulated MMP13^+/+^ mice compared to MMP13^−/−^ mice. Consequently, it is possible that MMP7 contributes to the observed difference in TNF activation in MMP13^+/+^ mice. However, the fact that MMP13 inhibition results in lower TNF levels, argues against this. Rather, this difference in MMP7 expression is likely a reflection of the increased Paneth cell activation, which is observed in the MMP13^+/+^ mice, which is a consequence of the observed LPS-induced mucus loss.

It was recently shown that TNF can affect tight junctions via the induction caveolin-dependent endocytosis of tight junction proteins (Marchiando et al, 2010). In agreement with this, we observed that caveolin-1 translocated from the lipid raft to the non-lipid-raft fraction after LPS challenge in MMP13^+/+^, but not in MMP13^−/−^ mice. In addition, by IEM and immunofluorescence, we observed severe effects of LPS on the subcellular localization of ZO-1, which is crucial for clustering of claudins with the actin cytoskeleton, again only in the MMP13^+/+^ mice. It is believed that ZO-1 forms a direct link between actin and the transmembrane tight junction proteins. Consequently, translocation of ZO-1 and thereby the associated contraction of cytoskeletal actin is thought to play an important role in the regulation of epithelial barrier function (Umeda et al, 2006). We did not observe LPS-dependent effects on the total protein level of different tight junction proteins, such as ZO-1, occludin and claudin-1, but it is known that it is mainly not the expression level but the expression pattern of tight junction proteins that determines epithelial permeability (Iwaya et al, 2012).

Not only the epithelial cell layer itself, but also the overlying mucus barrier, which consists of large glycoproteins called mucins, is extremely important for the intestinal barrier function. This is underlined by the observation that mice deficient in Muc2, the most abundant mucin in the small intestine, develop spontaneous intestinal inflammation (Van der Sluis et al, 2006). *In vitro*, we observed a TNF-dependent up-regulation of Muc2 gene expression in goblet cells, which is in agreement with literature where it was described that incubation of goblet cells with TNF induces mucin secretion within the first 24 h (Smirnova et al, 2001). Additionally, TNF injection *in vivo* resulted in a loss of goblet cells containing mucus, determined by quantification of total mucus and MUC2 immunofluorescence. This shows that TNF induces not only expression of mucus but also its secretion, finally resulting in the depletion of mucus. Indeed, we observed an LPS-induced decrease in mucus in MMP13^+/+^ but not in MMP13^−/−^ mice. The depletion of mucus by pilocarpine treatment resulted in sensitization for LPS-induced shock both in MMP13^+/+^ and MMP13^−/−^ mice, which further strengthens the notion of the important role of the mucus layer in endotoxemia. Recently, it was reported that spontaneous mucus depletion in *Winnie* and *Eeyore* mice, created by random mutagenesis of the Muc2 gene, was associated with ER stress (Heazlewood et al, 2008). In agreement with this, we observed, but only in MMP13^+/+^ mice, LPS-induced evidence of ER stress in goblet cells (by TEM) and altered intestinal expression of spliced Xbp1 and BiP, both of which are involved in ER stress (Kaser et al, 2011). Disappearance of the mucus layer in MMP13^+/+^ mice allows the intestinal bacteria to interact with IECs and Paneth cells, resulting in increased local inflammation and activation of Paneth cells. In LPS-injected MMP13^+/+^ mice this was reflected in up-regulation of inflammatory genes, such as IL6 and iNOS, and Paneth-cell specific genes, such as RegIIIg and MMP7. RegIIIγ is an antibacterial agent present in the Paneth cells induced by the commensal microbes (Wlodarska & Finlay, 2010). The importance of the intestinal bacteria was further strengthened by the observation that sterile MMP13^+/+^ mice were more resistant than non-sterile MMP13^+/+^ mice to LPS-induced shock.

Both TNF and mucin might be selectively altered before epithelial cell damage in DSS-induced colitis (Dharmani et al, 2011), one of the most comprehensively tested models of colitis that mimics the clinical and histological features of human UC (Yan et al, 2009). The absence or inhibition of TNF activity ameliorates disease progression in different experimental IBD models and in human patients. Treatment with a TNF neutralizing antibody, a locally active TNF inhibitor, antisense oligonucleotides and siRNA molecules specific for TNF were shown to be effective in the DSS-induced colitis model (Dharmani et al, 2011; Murthy et al, 2002; Myers et al, 2003; Ocampo et al, 2012). As expected, we observed that MMP13^−/−^ mice were less sensitive to DSS-induced colitis than MMP13^+/+^ mice and that this was correlated with reduced bioactive TNF levels. However, the protection was moderate, especially since treatment with a TNF-neutralizing antibody markedly reduces the DSS-induced clinical score (Dharmani et al, 2011). This can be due to the fact that MMP13 is not the only important TNF sheddase in this model or because other processes are also involved. Indeed, the chemical DSS model is of great value for a better understanding of acute inflammatory disease processes in IBD, but during the recovery phase, also other processes such as wound healing play an important role. Wound healing is known to be delayed in MMP13^−/−^ mice (Hartenstein et al, 2006). It has been suggested that altered sensitivity of genetically modified mice to DSS must be viewed in the context of epithelial cell injury and repair and should not be interpreted as a function of disrupted tight junction permeability alone (Brown & Mayer, 2007; Williams et al, 2001).

Although we do not exclude that also other MMP13 substrates play a role in the observed LPS resistance, we proved that the observed phenotype is highly dependent on TNF activity. Indeed, anti-TNF treatment of endotoxemic mice resulted in reduction of mortality, intestinal permeability and mucus containing goblet cell loss and TNFR1^−/−^ mice were protected against LPS-induced lethality.

**Figure 7:**
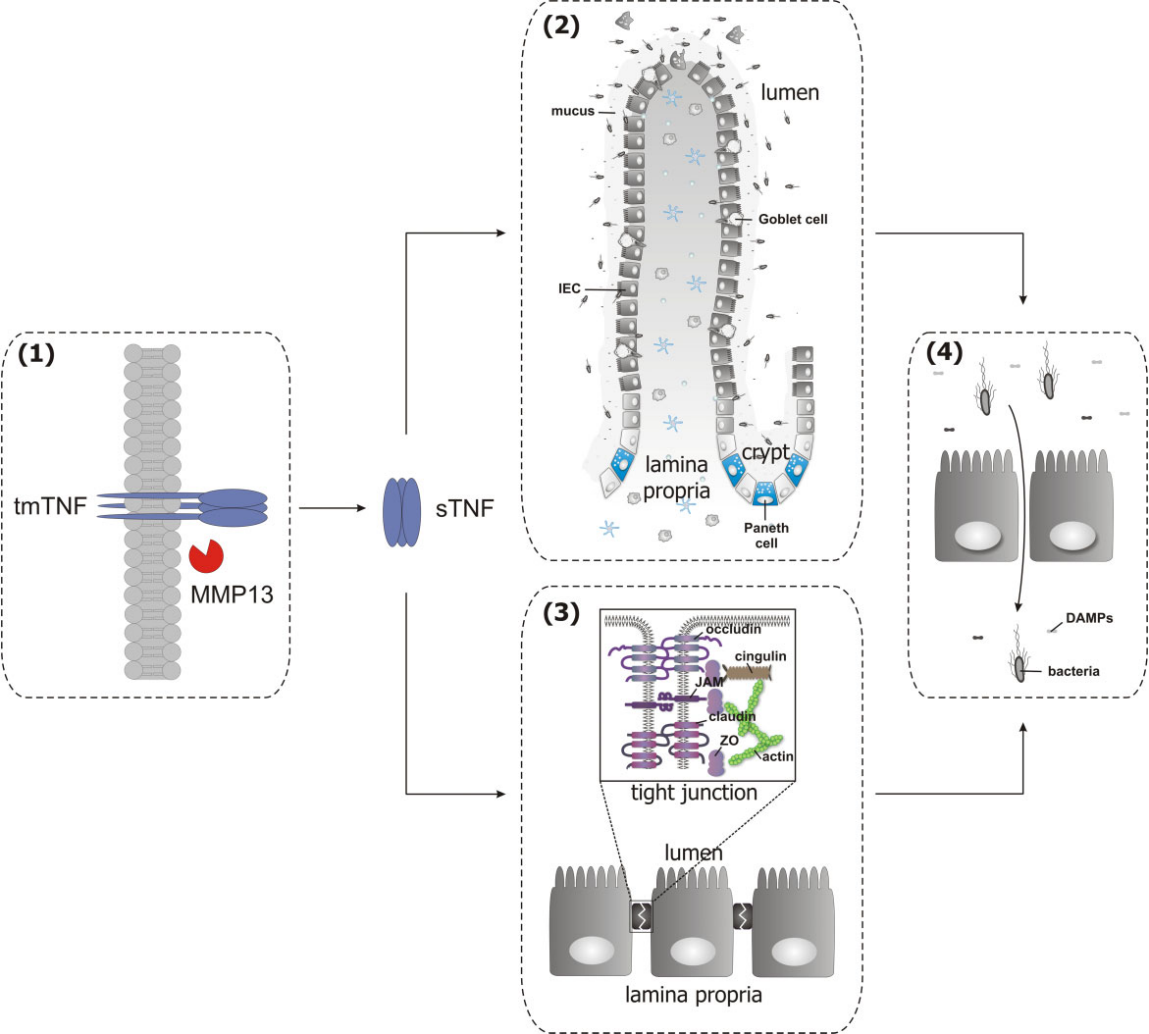
Effect of MMP13 on intestinal barrier dysfunction LPS-induced shock and DSS-induced colitis result in MMP13 up-regulation in the gut. This leads to MMP13-mediated shedding of transmembrane-bound TNF and release of bioactive, soluble TNF (1). Subsequently, sTNF induces goblet cell mucin expression and secretion and eventually results in ER-stress, which leads to mucus depletion and increased interaction of bacteria with IECs and Paneth cells (2). Additionally, TNF induces caveolin-dependent endocytosis, resulting in destabilization of tight junctions. This is associated with loss of tight junction functionality and increased intestinal permeability (3). Consequent leakage of intestinal components increases systemic inflammation, which leads to organ damage and eventually to death (4).

In conclusion (Fig 7), LPS-induced shock and DSS-induced colitis induce MMP13 up-regulation in the gut. This results in MMP13-mediated shedding of transmembrane-bound TNF and release of bioactive, soluble TNF. Subsequently, sTNF induces mucin expression and secretion by goblet cells, and eventually ER-stress, which results in mucus depletion and increased interaction of bacteria with IECs and Paneth cells. Additionally, TNF induces caveolin-dependent endocytosis, which destabilizes tight junctions. This causes loss of tight junction functionality and increased intestinal permeability. Consequent leakage of intestinal components increases systemic inflammation, which leads to organ damage and eventually to death.

Our results suggest that MMP13 is a potential therapeutic target for treatment of inflammatory disorders associated with TNF-dependent dysfunction of the intestinal barrier, such as sepsis and IBD. Unfortunately, the clinical use of pharmacologic MMP inhibitors has been hampered by their lack of specificity (Dorman et al, 2010). A major goal should be to identify compounds targeting MMP13 without interfering with other MMPs, not only for the previously suggested treatment of joint diseases (Takaishi et al, 2008), but as shown by our data also for the treatment of pathologies such as sepsis and IBD.

## MATERIALS AND METHODS

### Animals

C57BL/6J MMP13^−/−^ mice (Inada et al, 2004) were housed in an SPF animal facility with *ad libitum* access to food and water. We used both male and female mice (8–12 weeks old). All experiments were approved by the ethics committee of the Faculty of Science of Ghent University.

### Endotoxemia model and TNF, pilocarpine and anti-TNF injections

Mice were injected intraperitoneally (*i.p*.) with LPS from *Salmonella enterica* serotype *abortus equi* (Sigma; LD_100_ in MMP13^+/+^ mice: 17.5 mg/kg body weight) or with recombinant mouse TNF (LD_100_ in MMP13^+/+^ mice: 1.25 mg/kg body weight) produced in *Escherichia coli* and endotoxin-free purified in our lab. In the experiments on antibiotic-treated mice, the mice were injected with 12.5 mg/kg LPS intravenously (*i.v*.) instead of *i.p*. to avoid injection into the swollen caecum. In the pilocarpine experiments, MMP13^+/+^ mice were injected with 200 mg/kg pilocarpine 45 min prior to a sublethal LPS injection (*i.p*.; 6 mg/kg body weight). For MMP13^−/−^ mice, 17.5 mg/kg body weight LPS (*i.p*.) was used. Control animals received injections of PBS. Rectal bodytemperature was measured at different times after challenge. Mice were bled by heart puncture, and serum or EDTA plasma was prepared and stored at −20°C. Anti-TNF (etanercept; 400 µg) was administered to the mice 30 min prior to LPS injection.

### DSS model

Acute colitis was induced by addition of 2% DSS (dextran sulfate sodium; 36–50 kD; MP Biomedicals) to the drinking water for 5 days. Body weight, occult or gross blood lost per rectum, and stool consistency were determined daily. Faecal blood was determined using Hemoccult SENSA (Beckman Coulter) analysis. The baseline clinical score was determined on day 0. No weight loss was scored as 0, weight loss of 1–5% below baseline as 1, 5–10% as 2, 10–20% as 3, and >20% as 4. For bleeding, a score of 0 was assigned for no blood, 2 for positive Hemoccult, and 4 for gross bleeding. For stool consistency, a score of 0 was assigned for well-formed pellets, 2 for pasty stool, and 4 for liquid stool. These scores were combined and divided by three, resulting in a total clinical score ranging from 0 (healthy) to 4 (maximal colitis). To eliminate any diagnostic bias, mice were scored blindly. Postmortem, the entire colon was removed from caecum to anus, its length was measured as an indicator inflammation, and colon samples were taken for RNA isolation, protein analysis and for preparing PFA-fixed, paraffin-embedded sections.

### MMP13 inhibitor experiments

Four days after 4 ml of 3% thioglycollate injection, macrophages were isolated by peritoneal lavage and two million cells were plated in six-well plates. Four hours later culture medium was refreshed and cells were stimulated with LPS (100 ng/ml) in the absence or presence of MMP13 inhibitor (1 nM; Calbiochem) and medium was collected after different time points. For the explant experiments, mice were injected with LPS (17.5 mg/kg) and ileum was harvested 15 min later. Flushed ileum pieces of 1.5 cm were incubated *ex vivo* in the presence or absence of different concentrations MMP13 inhibitor (10 nM and 100 nM; Calbiochem).

### Caecal ligation and puncture model

Severe sepsis was induced in isoflurane anesthetized mice by ligation of the cecum, followed by twice puncturing with a 21-gauge needle as described earlier (Rittirsch et al, 2009). All animals received two doses of antibiotic therapy i.p. (ceftriaxone 25 mg/kg and metronidazole 12.5 mg/kg) at 9 and 24 h after CLP.

### Depletion of commensal intestinal bacteria

For antibiotic-mediated depletion of commensal bacteria, the drinking water was supplemented with 200 mg/L ciprofloxacin (Sigma–Aldrich), 1 g/L ampicillin (Sigma–Aldrich), 1 g/L metronidazole (Sigma–Aldrich) and 500 mg/L vancomycin (Labconsult). After 2 weeks, the presence of colonic microflora was determined by culturing faecal samples in brain heart infusion (BD Biosciences) and in thioglycollate medium (Sigma–Aldrich).

### Real time qPCR

Organs were stored in RNALater (Ambion) and RNA was isolated by using the RNeasy Mini Kit (Qiagen). cDNA was synthesized by the iScript cDNA Synthesis Kit (BioRad). Real time PCR was performed on the Light Cycler 480 system (Roche) using the LightCycler 480 SYBR Green I Master (Roche). Expression levels were normalized to the expression of the two most stable housekeeping genes, which were determined for each organ using the geNorm Housekeeping Gene Selection Software (Vandesompele et al, 2002).

### Cytokine/chemokine measurements

Quantification of cytokines and chemokines (in pg/ml) in serum was performed using the Bio-Plex cytokine assays (Bio-Rad), according to the manufacturer's instructions. TNF bioactivity (in U/mg tissue) was determined by using the L929 bioassay system.

### Intestinal permeability

FITC-labeled dextran (4 kDa, Sigma) was administered to mice by gavage at 150 mg/kg body weight. Five hours later, blood obtained by heart puncture was collected in EDTA-coated tubes (Sarstedt) and plasma was prepared. Leakage of FITC-labeled dextran into the circulation was determined by measurement of the plasma fluorescence (*λ*_ex_/*λ*_em_ = 488/520 nm). Values were normalized to the lowest value.

### Histopathology of small intestine

Tissues were fixed with 4% PFA, embedded in paraffin, sectioned at 4 µm, dewaxed and stained. After hematoxylin (Fluka) and eosin (Merck) staining, sections were mounted and the degree of damage was evaluated on entire organ sections by four neutral observers in a blinded fashion. Intestinal damage is characterized by decreased villus height, epithelial cell death at the villus top, and loss of mucus layer and goblet cells. Taking into account all histological features, a damage score ranging from 0 (normal) to 4 (abnormal) was given to each mouse. Alcian blue staining was performed by incubating the ileum sections for 30 min in 0.1% Alcian blue, followed by two washing steps and counterstaining with nuclear fast red for 5 min. Finally, sections were rehydrated and mounted. The amount and size distribution of Alcian blue positive goblet cells was measured automatically by using the Volocity software (Perkin–Elmer).

### Immunostaining

Tissues were fixed with 4% PFA, embedded in paraffin, sectioned at 4 µm, dewaxed and stained. For immunofluorescent labelling, sections were boiled in 10 mM sodium citrate buffer for antigen retrieval, incubated for 1 h in blocking buffer (10 mM Tris–HCl pH 7.4, 0.1 M MgCl_2_, 0.5% Tween20, 1% BSA and 5% serum), and incubated overnight with anti-BiP (1/40; C50B12, Cell signaling) or anti-MUC2 antibody (1/200; sc-15334, Santa Cruz). After rinsing with TBS/0.1% Tween-20, sections were incubated with fluorescently labeled secondary antibody DAR555 (1/400; Invitrogen). After another washing step, sections were counterstained with DAPI and mounted. Fluorescent images were taken by a laser scanning confocal microscope (Leica TCS SP5).

### Transmission electron microscopy (TEM)

The excised ileum was fixed in a solution of glutaraldehyde and formaldehyde (0.3%/2.5% for morphological EM analysis and 2.5%/4% immune-EM (IEM)) dissolved in 0.1 M sodium cacodylate buffer containing 20 mg/100 ml CaCl_2_. Fixed specimens were dehydrated through a graded ethanol series and embedded in Spurr's resin for morphological EM analysis or in LR White's resin for IEM. Ultrathin sections of a gold interference colour were cut using an ultramicrotome (Leica EM UC6) and sections were collected on formvar-coated copper slot grids. For IEM, grids were incubated with blocking solution (5% BSA, 1% FSG in PBS) for 20 min, followed by thorough washing with PBS/1% BSA. Next, sections were incubated for 2 h with anti-ZO-1 (1/5; 617300, Life Technologies) diluted in PBS/1% BSA. After another washing step, grids were incubated for 30 min with protein A-10-nm gold (PAG10nm; Cell Biology Department, Utrecht University) and with PBS/0.1% BSA, PBS, and double-distilled water. Control experiments consisted of treating sections with PAG10nm alone. Finally, sections were post-stained with uranyl acetate for 40 min and lead citrate for 7 min in a Leica EM AC20 and viewed with a transmission electron microscope (JEOL 1010; JEOL, Tokyo, Japan).

### Tight junction protein analysis

The excised ileum was washed with ice-cold PBS, cut open longitudinally, and mucosal samples were obtained by scraping to enrich for villi. Mucosal scrapings were homogenized in lysis buffer (50 mM Tris, 25 mM KCl, 5 mM MgCl_2_.6H_2_O, 2 mM EDTA, 40 mM NaF, 4 mM Na_3_VO_4_, pH 7.4) containing 1% Triton X-100 and a protease inhibitor mixture solution. For total protein analysis, samples were centrifuged for 15 min at maximal speed and supernatant was collected. Tight junction membrane microdomains were isolated according to a previously described method (Li et al, 2008). The homogenized samples were mixed with an equal volume of 80% sucrose in lysis buffer and loaded at the bottom of an ultracentrifuge tube. A discontinuous sucrose gradient was layered on top of the sample by layering 30, 25, 20 and 5% sucrose (2 ml each). The gradients were ultracentrifuged (250,000 g, 18 h at 4°C) in a Ti90 rotor in an Optima L-80XP ultracentrifuge (Beckman). Ten 1-ml fractions were collected from the top of each tube. Protein concentration was determined by BCA assay (Pierce) and samples were analysed by Western blot analysis. Blots were incubated overnight with anti-actin (1/10,000; 691002, MP Biomedicals), anti-claudin-1 (1/5000; 519000, Life Technologies), anti-occludin (1/250; 422400, Life Technologies), anti-ZO-1 (1/1000; 617300, Life Technologies) or anti-caveolin (1/500; ab2910, AbCam) antibody overnight at 4°C, followed by 1 h incubation with IRDye antibody (1/10,000; Westburg) at room temperature. Immunoreactive proteins were visualized and quantified using the Odyssey™ Infrared Imaging System and Odyssey software as described by the manufacturer (Li-Cor).

### Cell culture

HT29-MTX goblet cells were a kind gift from Dr. Thécla Lesuffleur (INSERM UMR S 938, Paris, France) (Lesuffleur et al, 1993) and were grown in DMEM supplemented with 25 mM glucose and 10% FBS. Cells were seeded in six-well plates and 21 days later, 2000 U/ml recombinant human TNF was added and samples were collected at different times, followed by RNA isolation. Recombinant human TNF was produced in *E. coli* and endotoxin-free purified.

### Quantification of total goblet cell mucus

(Kitagawa et al, 1986). The excised ileum was soaked for 2 h in 2 ml of 0.1% Alcian blue, dissolved in 0.16 M sucrose buffered with 0.05 M sodium acetate (pH 5.8). Uncomplexed dye was removed by two successive washes in 2 ml of 0.25 M sucrose for 15 and 45 min. Next, the mucus-complexed dye was extracted by treatment with laxative (30% disodium octyl sulfosuccinate) for 2 h. After centrifugation (3000 rpm for 10 min), the optical density of the extracted solution was read at 620 nm and the concentration of the extracted Alcian blue was calculated in comparison with a calibration curve obtained from known concentrations of Alcian blue solutions.

**The paper explained**

PROBLEM:

Sepsis is a highly mortal, inflammatory disease initiated by an infection and associated with systemic inflammation and organ dysfunction. The prevalence is high and current treatments are ineffective and mainly supportive. MMPs are important inflammatory mediators during sepsis, as well as inflammation at the level of the gut, but the link between these two has not been clearly established.

RESULTS:

We found that mice deficient for the collagenase MMP13 strongly resist sepsis and are protected against sepsis-induced gut permeability, goblet cell depletion, as well as induction of ER stress and tight junctions dysfunction in the small intestine. *In vitro* and *in vivo* experiments revealed that MMP13 is able to cleave membrane bound TNF, which is the initiator of the observed intestinal inflammation. Similarly, we also revealed a detrimental role for MMP13 in the TNF-dependent disease colitis.

IMPACT:

We propose MMP13 as a novel drug target for diseases in which TNF-induced damage to the gut is essential, such as sepsis and colitis.

### IL-FABP ELISA

Concentrations of ileal fatty acid binding protein in plasma were determined by a standard enzyme-linked immunosorbent assay (ELISA) for mouse IL-FABP according to manufacturer's instructions (Hycult Biotechnology).

### LDH activity

LDH activity in plasma was measured by the CytoTox 96 Assay (Promega), according the manufacturer's instructions.

### ProTNF cleavage assay

Recombinant MMP13 (511-MM-010, R&D) was diluted to 100 µg/ml in assay buffer (50 mM Tris, 10 mM CaCl_2_, 150 mM NaCl, 0.05% Brij-35, pH 7.5) and activated by incubation for 2 h at 37°C in the presence of 1 mM APMA. Recombinant proTNF fusion protein (1012-PS-010, R&D systems) was diluted in 50 mM Tris pH 8 at a concentration of 200 µg/ml. ProTNF was incubated with activated MMP13 at 37°C in a 1:1 ratio (w:w) and samples were taken at different times points. Reaction was stopped by addition of loading buffer followed by snap freezing. As a control for self-cleavage, proTNF and activated MMP13 samples were treated similarly, but without co-incubation. Equal amounts of sample were loaded on a 15% SDS–PAGE. Control samples (proTNF and MMP13 alone) were visualized by silver staining. Samples of co-incubated proTNF and MMP13 were analysed by western blot. Blots were incubated overnight with anti-TNF antibody (1/1000; SAB4502982, Sigma–Aldrich) followed by 1 h incubation with IRDye 800CW goat anti-rabbit IgG antibody (1/10,000) at room temperature. Immunoreactive proteins were visualized using the Odyssey™ Infrared Imaging System and Odyssey software as described by the manufacturer (Li-Cor).

### In-gel stable-isotope labelling and LC-MS/MS analysis

(Asara et al, 2006). The gel bands containing full length and possible TNF fragments upon cleavage by MMP13 were excised from the coommassie-stained SDS–PAGE and washed consecutively with water (Milli-Q purified, Millipore), water/acetonitrile (1/1, with acetonitrile HPLC grad; Biosolve) and acetonitrile, each for 15 min. After these washing steps, the gel bands were vacuum dried. Each gel band was then re-swollen in 1 mg NHS-trideutero-acetate (synthesized in-house according to (Staes et al, 2011)) dissolved in 100 µl 50 mM triethylammonium bicarbonate (Sigma–Aldrich) pH 8. Trideutero-acetylation of primary amines, *i.e*., ε-amines of lysines and α-amines of free (neo)N-termini, was allowed for 1 h at 30°C, followed by washing the gel bands again with water followed by acetonitrile, each for 15 min and vacuum dried. These trideutero-acetylation and washing steps were once repeated in order to reach maximal trideutero-acetylation. Subsequent washing the gel bands with 50 mM ammonium bicarbonate (Sigma–Aldrich) quenches any remaining NHS-ester, after which the gel bands were vacuum dried. Hydroxylamine (2 µl, 50 wt%; Sigma–Aldrich) in 100 µl 50 mM ammonium bicarbonate was added to each gel band for 20 min at 30°C in order to reverse possible *O*-trideutero-acetylation of Ser, Thr and Tyr. Samples were washed consecutively for 15 min with 50 mM ammonium bicarbonate and acetonitrile and vacuum dried. Each gel band was re-swollen with 10 µl 50 ng sequence grade trypsin (Promega Corporation). Gel bands were completely immersed in 50 mM ammonium bicarbonate and trypsin digestion was allowed overnight at 37°C. Two microliters of 100% formic acid was added to deactivate trypsin, the peptide mixtures were vacuum dried and re-dissolved in 20 µl of 0.1% trifluoroacetic acid (Biosolve) in 2% acetonitrile.

The peptide mixtures were then introduced into an LC-MS/MS system, the Ultimate 3000 RSLC nano (Dionex) in-line connected to an LTQ Orbitrap Velos (Thermo Fisher Scientific) for peptide identification. Sample mixture (2.5 µl) was loaded on a trapping column (made in-house, 100 µm internal diameter (I.D.) × 20 mm length, 5 µm C18 Reprosil-HD beads; Dr. Maisch). After flushing from the trapping column, the sample was loaded on a reverse-phase column (made in-house, 75 µm ID × 150 mm, 5 µm C18 Reprosil-HD beads; Dr. Maisch). Peptides were separated with a 30 min linear gradient from 2% solvent A (0.1% formic acid) to 50% solvent B (0.1% formic acid in 80% acetonitrile) at a flow rate of 300 nl/min followed by a wash with solvent B.

The mass spectrometer was operated in data-dependent mode, automatically switching between MS and MS/MS acquisition for the 10 most abundant peaks in a MS spectrum. Full scan MS spectra were acquired in the Orbitrap at a target value of 1E6 with a resolution of 60,000. The 10 most intense ions were isolated for fragmentation in the linear ion trap, with a dynamic exclusion of 20 s and fragmented after filling the ion trap at a target value of 1E4 ion counts. From the MS/MS data in each LC run, Mascot Generic Files were created using the Distiller software (version 2.4.2.0, Matrix Science, http://www.matrixscience.com/Distiller). These peak lists were then searched with the Mascot search engine (Matrix Science) using the Mascot Daemon interface (version 2.3, Matrix Science). Spectra were searched against the Swiss-Prot database (version 2012_05 of the UniProtKB/Swiss-Prot protein database) restricted to human proteins concatenated with the TNFα part of the fusion protein. Variable modifications were set to methionine oxidation, pyro-glutamate formation of N-terminal glutamine, S-propionamide formation of cysteines, acetylation of the peptide N-terminus and tri-deuteroacetylation of the peptide N-terminus. Fixed modifications were set to tri-deuteroacetylation on lysine. Mass tolerance on peptide ions was set to 10 ppm (with Mascot's C13 option set to 1), and to 0.5 Da for peptide fragment ions. The peptide charge was set to 1+, 2+, 3+ and instrument setting was put on ESI-TRAP. Enzyme was set to semi-ArgC/P, allowing for 1 missed cleavage, and cleavage was also allowed when arginine is followed by proline. Only peptides that were ranked one and scored above the threshold score, set at 99% confidence, were withheld. All further data management was done by ms_lims (Helsens et al, 2010).

### Statistical analysis

Data are presented as means ± SEM. Data were analysed with an unpaired Mann–Whitney *U*-test, unless mentioned differently. Survival curves were compared using a log-rank test. Significances were calculated for differences from the corresponding 0 h time point and/or between MMP13^+/+^ and MMP13^−/−^ mice, as indicated (*, 0.01 ≤ *p* < 0.05; **, 0.001 ≤ *p* < 0.01; ***, *p* < 0.001).

## Supplementary material

As a service to our authors and readers, this journal provides supporting information supplied by the authors. Such materials are peer reviewed and may be re-organized for online delivery, but are not copy-edited or typeset. Technical support issues arising from supporting information (other than missing files) should be addressed to the authors.
