# Supplementary material for: Matrix metalloproteinase 13 modulates intestinal epithelial barrier integrity in inflammatory diseases by activating TNF
Source: EMBO Mol Med. 2013 May 30;5(7):932–48. doi: 10.1002/emmm.201202100 (PMC3721470; doi:10.1002/emmm.201202100)
Supplement: Supplementary file 1 [file emmm0005-0932-SD1.pdf]

EMBO Molecular Medicine

# Matrix metalloproteinase 13 modulates intestinal epithelial barrier integrity in inflammatory diseases by activating TNF

Roosmarijn E. Vandenbroucke, Eline Dejonckheere, Filip Van Hauwermeiren, Sofie Lodens, Riet De Rycke, Elien Van Wonterghem, An Staes, Kris Gevaert, Carlos López-Otin, and Claude Libert

*Corresponding author: Claude Libert, Ghent University/VIB, Belgium*

---

**Review timeline:**

| | |
|---|---|
| Submission date: | 29 September 2012 |
| Editorial Decision: | 16 November 2012 |
| Revision received: | 15 March 2013 |
| Editorial Decision: | 08 April 2013 |
| Revision received: | 09 April 2013 |
| Accepted: | 11 April 2013 |

---

## Transaction Report:

(Note: With the exception of the correction of typographical or spelling errors that could be a source of ambiguity, letters and reports are not edited. The original formatting of letters and referee reports may not be reflected in this compilation.)

*Editor: Natascha Bushati / Céline Carret*

1st Editorial Decision 16 November 2012

---

Thank you for the submission of your manuscript to EMBO Molecular Medicine. We have now heard back from the three referees whom we asked to evaluate your manuscript. You will see that they find the topic of your manuscript potentially interesting. However, they also raise significant concerns on the study, which should be addressed in a major revision of the manuscript.

Reviewer #1 and #2 both highlight that further data is required to strengthen the link between MMP13 and TNFalpha cleavage in vivo. Reviewer #1 notes that the involvement of other MMPs should be addressed. Referee #2 would like to see further characterisation of possible TNFalpha-independent phenotypes in MMP13-/- mice. Importantly, this reviewer also notes that the validity of the LPS model has been questioned and suggests using an alternative model. In addition s/he would like to see analysis of the DSS model beyond application of DSS.

We would like to ask you to focus on addressing the concerns of referees #1 and #2. In addition, if you have data on hand addressing the concerns of referee #3, we would strongly encourage you to include these in the manuscript.

Given the balance of these evaluations, we feel that we can consider a revision of your manuscript if you can convincingly address the issues that have been raised within the space and time constraints outlined below. We would like to point out that it is our policy to allow one major round of revision only and that it is therefore important to address the raised concerns at this stage.

Revised manuscripts should be submitted within three months of a request for revision. If your revision will have to exceed this time frame, please contact the editor. Please also contact the editor

as soon as possible if similar work is published elsewhere.

I look forward to seeing a revised form of your manuscript in due course.

***** Reviewer's comments *****

Referee #1 (General Remarks):

This is an elegant study showing that MMP13 contributes to TNF bioavailability and TNF-dependent intestinal inflammation and epithelial barrier disruption in an endotoxemia model resembling human sepsis. These conclusions are based on comparison between wild type and MMP13-deficient mice. In addition MMP13 is shown to be able to cleave TNF in vitro. However, there are a few points that need to be addressed.

Specific comments:

The study lacks evidence that MMP13 activity mediates TNF cleavage in vivo. Additional proof to support this hypothesis could be provided by using MMP13 inhibitors (e.g TIMP-1 or -2) or gene silencing by RNAi in experiments with ex vivo tissue explants or isolated cells (e.g. endothelial or leukocyte, see p.3) from the control and mutant mice.

Previously several other MMPs have been shown to cleave TNF in vitro (see p.8). Among them MMP7 was also found to be upregulated after LPS injection selectively in MMP13+/+ mice. How about the expression (or induction) of the other implicated MMPs? Since MMP13 could also act through proteolytic cascades, these issues and the relative significance of potential direct MMP13 cleavages should be addressed. Also here, proof from cell culture or tissue explant experiments would be helpful.

Minor:

-In Fig1G-J the time-course of TNF levels was clearly different from the others, which could be described in Results.
-Several graphs have text with too small font size (e.g. fig. 2F-G).
-Showing representative WBs in Fig. 3A-C would be nice for visual conclusions.
-Fig. 2M is mislabeled
-In Fig. 2B-C image of normal ileum or arrows explaining the "similar intestinal damage" would be helpful.
-The text labels are missing from the microscope image 2G.
-Results p. 6. "... sXbp1 mRNA (Figure 2K) and BiP protein (Figure 2 L-M) were increased in MMP13+/+ but not MMP13-/- mice."
Is the "relative expression" in fig. 2 K a comparison to the control values? In fig. 2 L-J there is no increase shown (no images of the starting point), only expression at 8h after LPS.
-Fig. 3 G. Only 1 row is shown for -LPS (MMP13 wt or - mice?).
Fig. 4 C. Are the arrow colors mixed in the figure or legend?

Referee #2 (Comments on Novelty/Model System):

As detailed in my report, the sepsis model (bolus injection of LPS) does not reflect the situation of the human disease. There are better models such as the cecal ligation and puncture model.

The IBD model (2% DSS) is only used at the initial phase (acute phase) but not during the recovery phase, which will yield additional important data.

© EMBO 

Referee #2 (General Remarks):

The manuscript describes a study with MMP13-/- mice. These mice are protected from a bolus injection of LPS, which proved to be lethal in wt animals. The authors attributed the protection to reduced goblet cell depletion, ER stress, enhanced intestinal permeability and tight junction destabilization in the gut. The authors found in addition that MMP13 was able to cleave TNF in vitro at sites adjacent to the natural TNF cleavage site used by ADAM17. Furthermore, the authors found that MMP13-/- mice are less affected during the acute phase of DSS colitis as compared to wt mice. The authors conclude that MMP13 is an additional TNF converting enzyme and hypothesize that lack of TNF cleavage is the reason why MMP13-/- mice are protected from death following LPS injection.

This is an interesting study, which indicates that MMP13 could be a therapeutic target for the treatment of septic conditions and inflammatory bowel disease. There are, however a couple of points, the authors need to address.

Major points:

1. The authors use bolus injection of LPS as a model for sepsis. The validity of this model has been questioned by many studies. The authors should consider to also use an alternative model such as the cecal ligation and puncture model, which more closely mimics the condition of the human disease.
2. The authors measure the increased mRNA expression of MMP13 upon LPS injection. Was this accompanied by an increase of MMP13 activity on the cell surface?
3. The time course of the cytokines and chemokines measured upon LPS injection is not uniform: some factors are not changed in the initial phase of observation but are decreased in the later phase; others are lowered throughout the obersation period. This should be commented and discussed by the authors.
4. The decrease in goblet cells in wt mice is rather small after 4 h; did the authors also measure after 20 h when the difference in barrier permeability was more pronounced (see Fig. 2E and 2A).
5. How did MMP13-/- mice react to LPS upon pretreatment with the mucus depleting agent pilocarpine? This experiment should be presented.
6. The authors argue that lack of TNF cleavage is responsible for the changes they observe in MMP13-/- mice. This has actually not been formally demonstrated in the study. Are the same changes (reduced goblet cell depletion, ER stress, enhanced intestinal permeability and tight junction destabilization in the gut) seen in wt mice upon TNF neutralization? It might well be that MMP13 exerts additional effects, which are relevant to survival upon LPS injection.
7. It has been shown some time ago (Robache-Gallea et al, 1995) that proteinase-3 (PR-3) can also efficiently cleave TNF in vitro and that the cleavage site used by this enzyme was just one amino acid away from the cleavage site used by ADAM17. Moreover, as described in the recent report by Le Gall et al (2009), also ADAM10 can cleave TNF when ADAM17 is absent. This should be mentioned and discussed in this manuscript. Do the authors have data to show that the loss of MMP13 can be compensated by other proteases?
8. In the DSS model, the authors chose to only analyze the first 5 days, i.e. the time when DSS was applied. In the DSS model these 5 days are followed by 5 days without DSS. During this time wound healing takes place. The authors should also study this time period since it is not unlikely that lack of MMP13 might have an effect during the recovery phase.
9. The authors mention the 'uncleavable TNF ', which has been used in the study described by Mueller et al. (1999). In this TNF , cleavage by ADAM17 has been abolished by introducing 3 point mutations close to the cleavage site. It would be interesting to know whether this mutant TNF is still cleaved by MMP13.
10. On p13 of the manuscript the authors write: 'The importance of the intestinal bacteria was further strengthened by the observation that sterile MMP13+/+ mice were more resistant than MMP13-/- mice to LPS-induced shock. This experiment is not shown in the manuscript. In Fig. 5E, only wt mice are shown.
11. For inflammatory bowel disease it has been shown in many studies that activation of the EGF receptor pathway and activation of the STAT3 pathway is important for wound healing upon DSS application. This should be discussed in view of the experiments presented in the current study.

Minor points:

1. The study by Le Gall et al (2009) should be taken into account for the discussion of cleavage of one substrate by several proteases. This study demonstrates that not only ADAM17 but also ADAM10 (in the absence of ADAM17) can efficiently cleave TNF . This might also apply to MMP13.
2. In Fig. 4D and Fig. 6D the authors define the activity of TNFa in U/mg tissue. They should also give the TNF levels in pg/ml as in Fig. 1H.
3. The different amounts of LPS used in different experiments should be commented and explained (p15).
4. Some references are not correctly formatted.

Referee #3 (Comments on Novelty/Model System):

The techniques used in this study are of very good quality. Similarly, the statistical analysis was done correctly.

The novelty of this study is medium because it had previously shown that:
-LPS increases paracellular permeability and bacterial translocation participating to sepsis.
-LPS induces the secretion of TNF
-TNF increases the paracellular permeability by caveolin mediating endocytosis and/or induction of MLCK expression and activity.

The novelty is the role of MMP13 in the cleavage of TNF.

3. The medical impact of this discovery is currently considered average but could be higher if inhibitors of MMP13 were available.

4. The models used in this study are adequate.

Referee #3 (General Remarks):

Matrix metalloproteinase 13 modulates intestinal epithelial barrier integrity in inflammatory diseases by activating TNF .

The group of Claude Libert provides an interesting manuscript showing that an induction of MMP13 expression and activity in response to LPS injection alters epithelial barrier function causing mice death and colitis severity induced by DSS infusion. Thus, MMP13-/- mice display a strong protection toward sepsis induced by LPS. This protection is linked to an absence or a reduction of the LPS ability to trigger goblet cell depletion, ER stress, permeability, and tight junction destabilization through a mechanism depending of the MMP13. The authors also reported that MMP13 is able to cleave pro-TNF into bioactive TNF supporting that MMP13 is an alternative pathway (in contrast with the TNF converting enzyme TACE) to mature TNF . Finally, similar mechanism was responsible for the observed protection of the MMP13-/- mice in a mouse model of DSS-induced colitis.
In general the paper is well written and this is a presentation of well performed experiments that are carefully interpreted. Nevertheless some controls are missing and the demonstration of the mechanism suffers of some weakness. Finally, there is no molecular data inside cell evidencing how the LPS induced MMP13 expression and activity. All of these criticisms are detailed below.

Major comments:
1) To explain the mechanism by which LPS increases the paracellular permeability, the authors have evidenced that TNF induces caveolin-dependent endocytosis of some of tight junction proteins. Nevertheless, the experience of cell fractionation is not enough to support such a conclusion. The

authors should demonstrate by using inhibitors of the different endocytosis pathways that caveolin is responsible for the endocytosis of tight junction protein.

2) The authors have evidenced that LPS increases the paracellular permeability by a mechanism involving caveolin endocytosis. However, a robust literature clearly evidenced that LPS alters paracellular permeability by a mechanism involving the myosin light chain kinase (MLCK) and not the endocytosis. How the authors explain this discrepancy? and it would be nice whether the role of the MLCK might be invalidated or validated. For that a treatment with MLCK inhibitors (ML-7, ML-9) should be performed.

3) To reinforce the role of the TNF in the death induced by LPS, a control is lacking. Although TNF treatment of MMP13-/- mice suppresses the protection toward the death induced by LPS, the authors should absolutely evidence that MMP13+/+ mice treated with anti-TNF antibodies present a better survival in response to LPS.

4) The authors evidenced that TNF secreted in response to LPS provokes mice death. However, we did not know which type of TNF receptors is involved? Is it the receptor type1 or 2?

5) The authors evidenced that TNF induces mucus secretion, goblet cell depletion as well as ER stress. In addition, the authors have shown that mucus depletion in the MMP13-/- mice induced by pilocarpine present a similar rate of mortality than observed in the MMP13+/+ mice treated with LPS. What is the mechanism by which TNF induces the depletion of mucus and goblet cells? Is it the induction of ER which triggers the depletion of mucus and goblet cells? If it is the case the authors should show that the inhibition of ER by tudca treatment ameliorates the survival rate. In addition, the authors have evidenced no apoptosis in response to LPS. What is the mechanism by which LPS decreases the number of Goblet cells? Finally, as BIP is an essential chaperon protein, it is very surprise that no staining of BIP was observed in MMP13-/- after LPS treatment. To reinforce the induction of ER, the protein expression of CHOP should be tested. Moreover, as CHOP is pro-apoptotic protein, this will allow the authors to exclude an involvement of apoptose.

6) The authors claim that the death of mice induced by LPS is linked with an increase of paracellular permeability and the depletion of both mucus and goblet cells. What is the most important parameter involved in the mice death? Is it the increase of paracellular permeability or the depletion of mucus and goblet cells? To test these hypotheses, it would be nice to treat the mice with inhibitors of the caveolin-endocytosis to block the increase of paracellular permeability and to monitor the mice death. In addition, the use of Math1flox/flox mice depleted for goblet cells could allow testing the involvement of goblet cell in the mice survival in response to LPS.

7) To reinforce the role of MMP13 in the response to LPS, the authors should test the expression and the activity of the TACE following LPS injection. In addition the use of KO mice for TACE expression would be more significant.

8) It is unfortunate that we have no data concerning the molecular mechanism by which LPS increase expression and activity of MMP13.

9) The authors will show that antibiotic treatment is effective in inducing decontamination or at least a significant reduction of the bacterial load to validate their results.

10) To reinforce the impact of TNF in the regulation of Muc2 expression, the quantification of protein level is absolutely required. At least, in HT29MTX model, only the mRNA level has been performed.

Minor comments:

1) The data concerning the apoptosis (TUNEL) are important and it should be including in the supplementary file.

2) Muc2 immunostaining is an important data and it should be including in the supplementary file.

3) Although the authors have performed immunostaining of BIP (qualitative approach), a quantitative approach such as western blot is required.

4) The picture of the western blot for ZO-1, Occludin and Claudin 1 should be including the figure 3.

5) In the figure 7, the authors present a synthetic schema of the mechanism by which LPS alters the homeostasis of the gut mucosa. However, the bacteria did not translocate across gut mucosa through tight junction, but across the cells (transcellular pathway). Moreover, the maximum aperture of the tight junctions is 120A. Please can you delete or move the arrow number 4.

1st Revision - authors' response 15 March 2013

Response to the editor

(1) '*Further data is required to strengthen the link between MMP13 and TNFa cleavage.*'

In the original manuscript we showed that:

- *In vitro*: recombinant MMP13 is able to cleave and activate proTNF.

- *In vivo*: we observed higher cleaved TNF levels in ileum lysates of MMP13$^{+/+}$ mice compared to MMP13$^{-/-}$ mice.

To further strengthen the link between MMP13 and TNF cleavage we made use of a commercially available and validated MMP13 inhibitor. Both in primary macrophages and in ileum explants, MMP13 inhibition lowers the levels of cleaved TNF (see rebuttal Fig. 1 & 2), which further proves that MMP13 cleaves TNF both *in vitro* and *in vivo*.

(2) '*The involvement of other MMPs should be addressed.*'

As suggested by both referee 1 & 2, we addressed the involvement of different MMPs, especially the ones that are believed to be involved in TNF cleavage. The effect of MMP13 on (the activation of) other MMPs is not easy to address since it is quite difficult to specifically detect protein expression or activity of individual MMPs. As an alternative, we studied MMP expression in unstimulated and stimulated conditions (see rebuttal Fig. 3 & Fig. 9) and this excludes the presence of compensatory mechanisms in the MMP13$^{-/-}$ mice.

(3) '*Further characterization of possible TNFa-independent phenotypes in MMP13$^{-/-}$ mice.*'

Although we do not exclude that also other MMP13 substrates play a role in the observed LPS resistance, we included new data in the revised manuscript that further prove that the phenotype is highly dependent on TNF activity. We addressed this issue by treating wild type mice with anti-TNF and studying LPS-induced mortality, intestinal leakage and mucus loss. In agreement with our suggested importance of TNF, anti-TNF treatment of endotoxemic mice resulted in reduction of mortality, intestinal permeability and mucus containing goblet cell loss (see rebuttal Fig. 8). Additionally, TNFR1$^{-/-}$ mice were protected against LPS-induced lethality (see rebuttal Fig. 11).

(4) '*The validity of the LPS model has been questioned: an alternative model?*'

Indeed, LPS is a good model to mimic sepsis, but it has its limitations. An alternative model, such as caecal ligation and puncture (CLP), is indeed interesting to confirm the importance of our findings. As shown in rebuttal Fig. 4, MMP13$^{-/-}$ mice showed significant protection against CLP-induced lethality.

(5) '*Analysis of the DSS model beyond application of DSS.*'

In the revised manuscript, we now included the clinical score of the DSS-treated mice until day 10 (see rebuttal Fig. 10).

(6) '*Focus on addressing the concerns of referees #1 and #2. In addition, if you have data on hand addressing the concerns of referee #3, we would strongly encourage you to include these in the manuscript.*'

We addressed all concerns of both referee #1 & #2 and most of the concerns of referee #3, as suggested.

Response to referee #1:

'*This is an elegant study showing that MMP13 contributes to TNF bioavailability and TNF-dependent intestinal inflammation and epithelial barrier disruption in an endotoxemia model resembling human sepsis. These conclusions are based on comparison between wild type and MMP13-deficient mice. In addition MMP13 is shown to be able to cleave TNF in vitro. However, there are a few points that need to be addressed.*'

*Major Comments:*

(1) '*The study lacks evidence that MMP13 activity mediates TNF cleavage in vivo. Additional proof to support this hypothesis could be provided by using MMP13 inhibitors (e.g. TIMP-1 or -2) or gene silencing by RNAi in experiments with ex vivo tissue explants or isolated cells (e.g. endothelial or leukocyte, see p.3) from the control and mutant mice.*'

We thank the referee for this suggestion. To address this, we made use of primary macrophages and an ileum explant system.

<u>Primary mouse macrophages</u> were isolated by peritoneal lavage, 4 days after 3% thioglycollate injection. Cells were plated and incubated with LPS, in the presence and absence of MMP13 inhibitor. Soluble TNF levels were analysed in the supernatant and this revealed reduced TNF levels in the presence of MMP13 inhibitor (rebuttal Fig. 1).

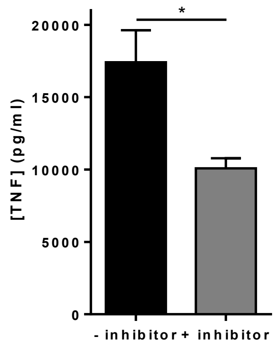


Fig. 1: TNF levels of *in vitro* LPS-stimulated macrophages incubated in the absence or presence of MMP13 inhibitor.

For the explant system, mice were injected with LPS and 1.5 cm ileal pieces were isolated 15 minutes later. The obtained ileum explants were incubated *ex vivo* in medium supplemented with or without MMP13 inhibitor and TNF levels were analysed 1 and 4 h later. As displayed in rebuttal Fig. 2, incubation with MMP13 inhibitor results in reduced TNF levels.

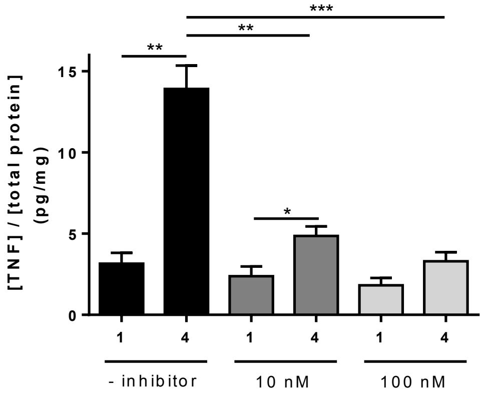


Fig. 2: TNF levels of LPS stimulated ileum explants incubated 1 and 4 hours *ex vivo* with (10 and 100 nM) and without MMP13 inhibitor.

We included both graphs: Fig. 4E and Fig. 4F of the revised manuscript.

(2) '*Previously several other MMPs have been shown to cleave TNF in vitro (see p.8). Among them MMP7 was also found to be up-regulated after LPS injection selectively in MMP13$^{+/+}$ mice. How about the expression (or induction) of the other implicated MMPs? Since MMP13 could also act through proteolytic cascades, these issues and the relative significance of potential direct MMP13 cleavages should be addressed. Also here, proof from cell culture or tissue explant experiments would be helpful.*'

Indeed, the observed difference in TNF between MMP13$^{+/+}$ and MMP13$^{-/-}$ mice is maybe not only caused by direct MMP13-dependent proTNF cleavage, but also other MMPs may be implicated. The effect of MMP13 on (the activation of) other MMPs is not easy to address since it is quite difficult to specifically detect protein expression or activity of individual MMPs. However, we did check if Adam17/TACE and MMPs implicated in TNF cleavage according to literature (MMP1, -2, -3, -9, -12, -14 and -15 [1-4]) are differentially expressed in ileum lysates of MMP13$^{+/+}$ mice compared to MMP13$^{-/-}$ mice (rebuttal Fig. 3).

In unstimulated conditions, we only observed a difference in MMP3 expression, which was lower in MMP13$^{-/-}$ mice compared to MMP13$^{+/+}$ mice. These data show that there is no compensation mechanism in the ileum of MMP13$^{-/-}$ mice that results in up-regulation of other MMPs.

After LPS stimulation, some MMPs showed down regulation, while other MMPs were up regulated. Only in case of MMP14 there was a significant difference: higher gene expression in MMP13$^{-/-}$ mice compared to MMP13$^{+/+}$ mice. Consequently, this cannot be an explanation for the lower TNF levels in the MMP13$^{-/-}$ mice.

However, in the original manuscript, we did show that LPS stimulation resulted in higher MMP7 expression in MMP13$^{-/-}$ compared to MMP13$^{+/+}$ mice. Consequently, it could be possible that MMP7 contributes to the observed cleaved TNF in the MMP13$^{+/+}$ mice *in vivo*. However, our explant data with the MMP13 inhibitor described above (rebuttal Fig. 1 and 2) argue against this. We included the data of MMP expression in unstimulated mice in the revised manuscript as supplemental material and discussed the possible role of MMP7 in the 'Discussion section' of the revised manuscript.

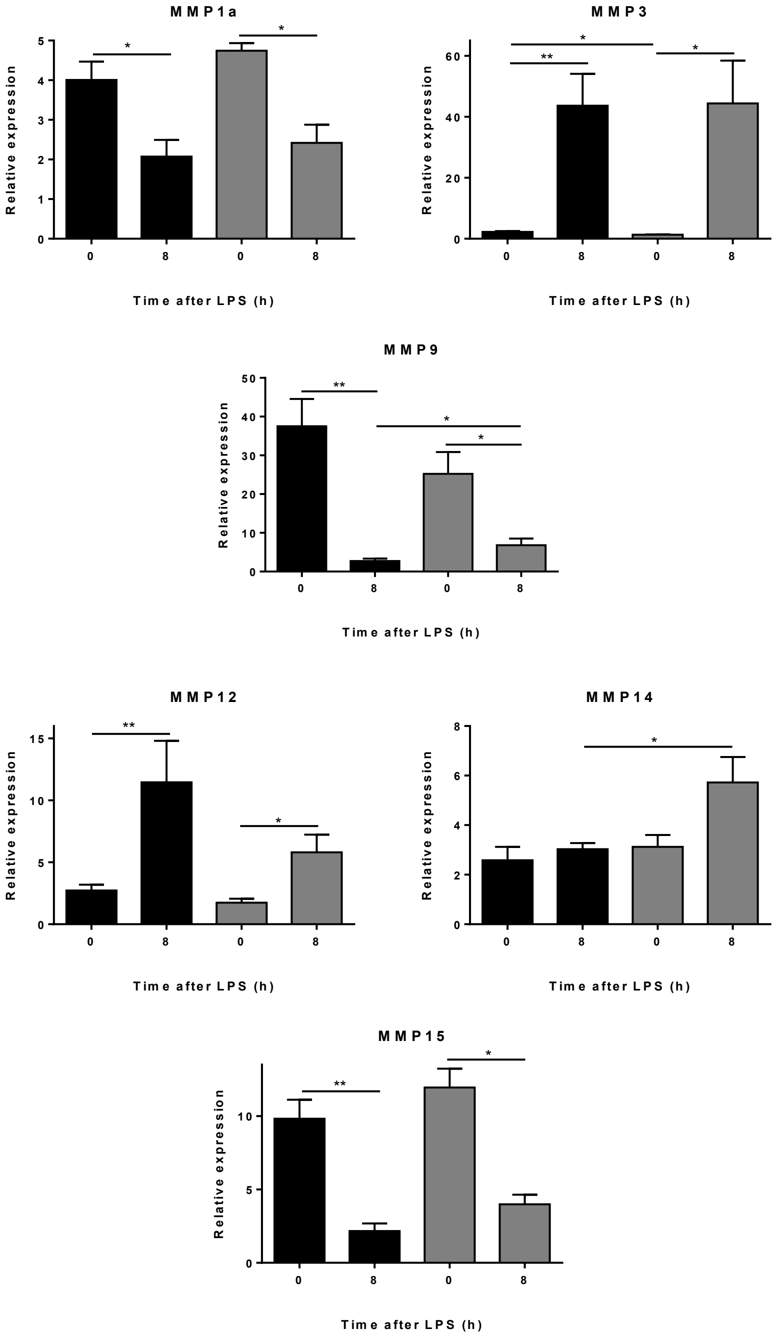

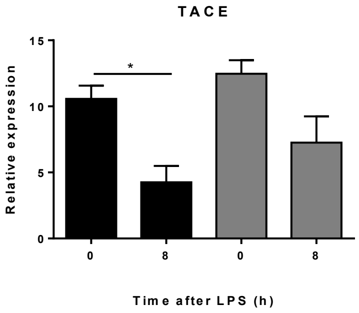


Fig. 3: MMP and TACE expression in ileum before and 8 h after LPS stimulation in MMP13$^{+/+}$ and MMP13$^{-/-}$ mice.

*Minor Comments:*

(1) '*In Fig1G-J the time-course of TNF levels was clearly different from the others, which could be described in Results.*'

As recently described in the paper of Bosmann et al. (2013), there are substantial differences in the absolute concentrations, time courses and clearance among distinct inflammatory mediators. Most mediators reach their maximum around 6-12 h, with the exception of a rapid increase of TNF after only 1 h, followed by a rapid decline. In the revised manuscript, we now described this in the 'Result section' in the revised manuscript, as suggested by both referee #1 and #2.

(2) '*Several graphs have text with too small font size (e.g. fig. 2F-G).*'

We agree and have adapted the text font size in several graphs.

*(3) 'Showing representative WBs in Fig. 3A-C would be nice for visual conclusions.'*

We included representative western blot images in Fig. 3A till 3C.

*(4) 'Fig. 2M is mislabelled'*

We corrected this mislabelling.

(5) '*In Fig. 2B-C image of normal ileum or arrows explaining the "similar intestinal damage" would be helpful.*'

We added arrows to indicate the intestinal damage and described the observations more in detail.

(6) '*The text labels are missing from the microscope image 2G.*'

We added text labels to the microscope image.

(7) '*Results p. 6. "... sXbp1 mRNA (Fig. 2K) and BiP protein (Fig. 2 L-M) were increased in MMP13+/+ but not MMP13-/- mice." Is the "relative expression" in fig. 2 K a comparison to the control values? In fig. 2 L-J there is no increase shown (no images of the starting point), only expression at 8h after LPS.*'

We indeed only displayed the 8 h time point, so we adapted the text according to the displayed figure into "Additionally, two markers for ER stress, namely spliced Xbp1 (sXbp1) mRNA (Fig. 2M) and BiP protein (Fig. 2 N-O) were higher in LPS-stimulated MMP13$^{+/+}$ compared to MMP13$^{-/-}$ mice. "

(8) '*Fig. 3 G. Only 1 row is shown for -LPS (MMP13 wt or - mice?). Fig. 4 C. Are the arrow colours mixed in the Fig. or legend?*'

We displayed a representative image of unstimulated MMP13$^{+/+}$ and MMP13$^{-/-}$ mice (Fig. 3G). We adapted the figure legend of Fig. 4C.

Response to referee #2:

'*As detailed in my report, the sepsis model (bolus injection of LPS) does not reflect the situation of the human disease. There are better models such as the cecal ligation and puncture model.*

*The IBD model (2% DSS) is only used at the initial phase (acute phase) but not during the recovery phase, which will yield additional important data.*

*The manuscript describes a study with MMP13$^{-/-}$ mice. These mice are protected from a bolus injection of LPS, which proved to be lethal in wt animals. The authors attributed the protection to reduced goblet cell depletion, ER stress, enhanced intestinal permeability and tight junction destabilization in the gut. The authors found in addition that MMP13 was able to cleave TNFa in vitro at sites adjacent to the natural TNFa cleavage site used by ADAM17. Furthermore, the authors found that MMP13-/- mice are less affected during the acute phase of DSS colitis as compared to wt mice. The authors conclude that MMP13 is an additional TNFa converting enzyme and hypothesize that lack of TNFa cleavage is the reason why MMP13$^{-/-}$ mice are protected from death following LPS injection.*

*This is an interesting study, which indicates that MMP13 could be a therapeutic target for the treatment of septic conditions and inflammatory bowel disease. There are, however a couple of points, the authors need to address.*'

*Major Comments:*

(1) '*The authors use bolus injection of LPS as a model for sepsis. The validity of this model has been questioned by many studies. The authors should consider to also use an alternative model such as the cecal ligation and puncture model, which more closely mimics the condition of the human disease.*'

Indeed, the ceacal ligation and puncture (CLP) model more closely mimics human sepsis, while a bolus LPS injection mimics the acute inflammatory phase without the presence of replicating bacteria. We applied the CLP model and found that MMP13$^{-/-}$ mice also show significant protection in this model compared to MMP13$^{+/+}$ mice. We included this graph: Fig. 1G in the revised manuscript.

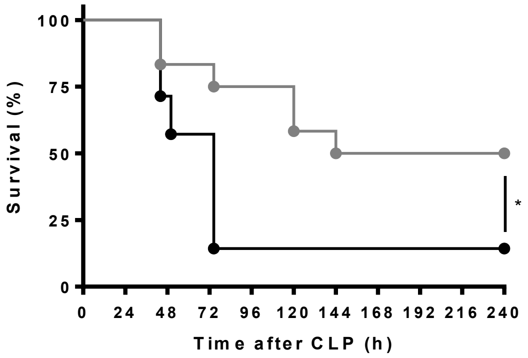


Fig. 4: Survival graph of $MMP13^{+/+}$ (black; n=7) and $MMP13^{-/-}$ (grey; n=12) mice subjected to CLP.

(2) '*The authors measure the increased mRNA expression of MMP13 upon LPS injection. Was this accompanied by an increase of MMP13 activity on the cell surface?*'

To address this question, we performed MMP13 immunostaining on ileum sections. This revealed MMP13 staining in epithelial cells and in (inflammatory?) cells in the lamina propria (Fig. 5A). Four hours after LPS injection (Fig. 5B), the cytoplasmic staining at the top of the villi shows reduced MMP13 staining, which suggests MMP13 secretion. In contrast, the amount of MMP13 positive lamina propria cells increases after LPS. These immunostainings are now included: Fig. 2A and 2B in the revised manuscript.

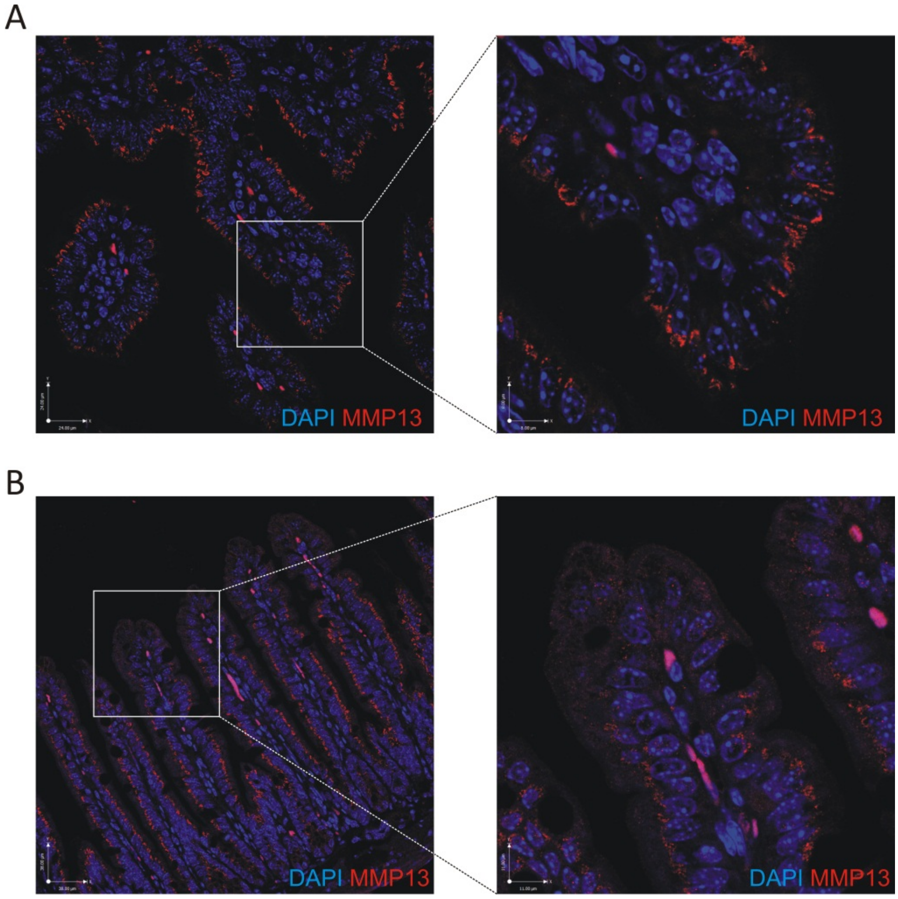

Fig. 5: MMP13 (red) immunostaining in the ileum of (A) unstimulated (B) LPS-stimulated mice (4 h).

(3) '*The time course of the cytokines and chemokines measured upon LPS injection is not uniform: some factors are not changed in the initial phase of observation but are decreased in the later phase; others are lowered throughout the observation period. This should be commented and discussed by the authors.*'

As recently described in the paper of Bosmann et al. (2013), there are substantial differences in the absolute concentrations, time courses and clearance among distinct inflammatory mediators. Most mediators reach their maximum around 6-12 h, with the exception of a rapid increase in TNF after only 1 h, followed by a rapid decline. We now described this in the Result section of the revised manuscript, as suggested by both referee #1 and #2.

(4) '*The decrease in goblet cells in wt mice is rather small after 4 h; did the authors also measure after 20 h when the difference in barrier permeability was more pronounced (see Fig. 2E and 2A).*'

Indeed, based on the observed difference in barrier permeability, one would expect more drastic differences in amount of mucus containing goblet cells at later time points. To address this, we isolated ileum samples 24 h after LPS stimulation and performed mucin-2 immunostaining. As shown in rebuttal Fig. 6, the MMP13$^{-/-}$ mice contain much more mucus containing goblet cells (Fig. 6B), in contrast to MMP13$^{+/+}$ mice (Fig. 6A). We included this observation in the revised manuscript as supplemental material.

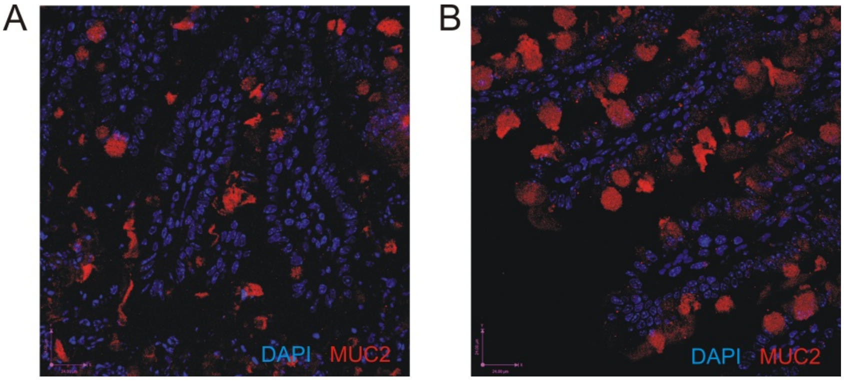


Fig. 6: Mucin-2 (red) immunostaining of ileum sections of (A) MMP13$^{+/+}$ and (B) MMP13$^{-/-}$ mice 24 h after LPS injection.

(5) '*How did MMP13$^{-/-}$ mice react to LPS upon pretreatment with the mucusdepleting agent pilocarpine? This experiment should be presented.*'

We thank the referee for this suggestion. Indeed, if the MMP13$^{-/-}$ phenotype is dependent on the presence of mucus, mucus depleted MMP13$^{-/-}$ mice should be more sensitive to LPS treatment. Indeed, mucus depletion sensitized MMP13$^{-/-}$ mice (rebuttal Fig. 7). We included this graph in the revised manuscript: Fig. 2I.

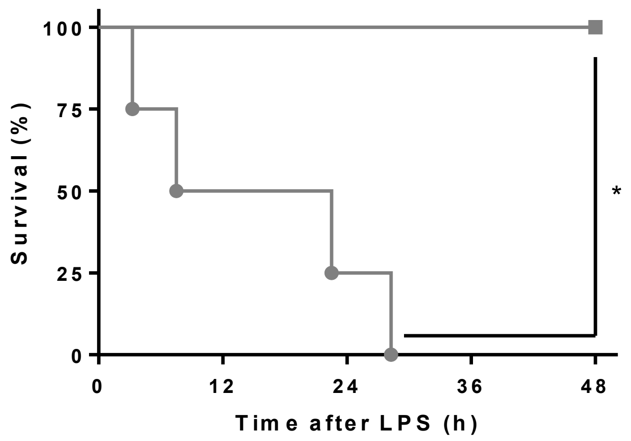


Fig. 7: Survival curve of LPS-injected untreated (square; n=4) and pilocarpine treated (circle; n=3) MMP13$^{-/-}$ mice.

(6) '*The authors argue that lack of TNFa cleavage is responsible for the changes they observe in MMP13$^{-/-}$ mice. This has actually not been formally demonstrated in the study. Are the same changes (reduced goblet cell depletion, ER stress, enhanced intestinal permeability and tight junction destabilization in the gut) seen in wt mice upon TNFa neutralization? It might well be that MMP13 exerts additional effects, which are relevant to survival upon LPS injection.*'

We thank the referee for this suggestion. Based on this question, we studied the effect of TNF neutralization, i.e. treatment with Etanercept, on LPS-induced lethality, intestinal permeability and loss of mucus containing goblet cells.

As shown in rebuttal Fig. 8A, TNF neutralization protects mice from LPS-induced lethality. In agreement with our hypothesis, the same treatment was associated with a reduction in LPS-induced intestinal permeability (rebuttal Fig. 8B). Moreover, mucin-2 staining of ileal sections revealed more mucus containing goblet cells anti-TNF treated mice compared to PBS treated mice after LPS challenge (rebuttal Fig. 8C and 8D). These results clearly indicate a strong similarity in the protective mechanism of anti-TNF treatment and absence of MMP13. Consequently, although we do not exclude the involvement of other MMP13 substrates, we strongly believe that these results suggest that the observed phenotype is mainly dependent on the MMP13 cleavage of TNF. All these data are incorporated in the revised manuscript: Fig. 5F-5G.

© EMBO

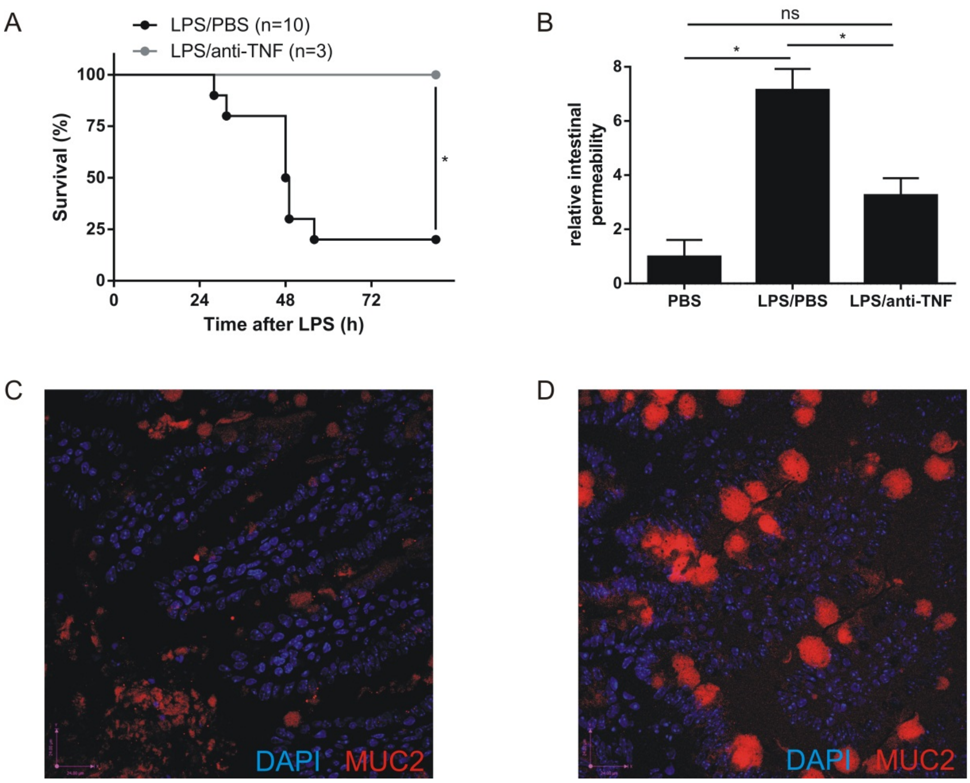


Fig. 8: (A) Anti-TNF treatment protects mice from LPS-induced lethality. (B) Anti-TNF treatment protects from LPS-induced permeability. Ileal sections of LPS challenged (C) PBS and (D) anti-TNF treated mice stained for mucin-2 (red) and nuclear staining (blue).

(7) '*It has been shown some time ago (Robache-Gallea et al, 1995) that proteinase-3 (PR-3) can also efficiently cleave TNFa in vitro and that the cleavage site used by this enzyme was just one amino acid away from the cleavage site used by ADAM17. Moreover, as described in the recent report by Le Gall et al (2009), also ADAM10 can cleave TNFa when ADAM17 is absent. This should be mentioned and discussed in this manuscript. Do the authors have data to show that the loss of MMP13 can be compensated by other proteases?*'

Although we cannot exclude this completely, we have no evidence that loss of MMP13 can be compensated by other proteases. To address this, we analysed gene expression in unstimulated ileal lysates. We focused on the proteases suggested by the referee (Adam10, Adam17 and PR-3) and some other MMPs, which might be involved in TNF cleavage. None of the analysed genes showed up regulation in MMP13$^{-/-}$ mice, only MMP3 gene expression seems to be reduced (see rebuttal Fig. 9). We discussed this now in the revised manuscript and added this as supplemental material.

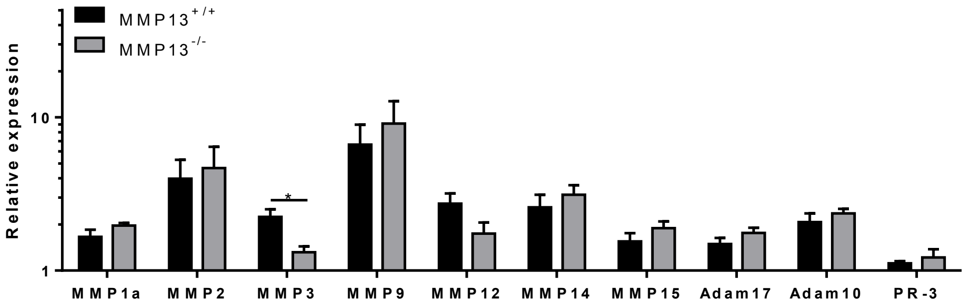

Fig. 9: MMP expression in unstimulated MMP13$^{+/+}$ (black) and MMP13$^{-/-}$ (grey) mice (n=4-5).

(8) '*In the DSS model, the authors chose to only analyse the first 5 days, i.e. the time when DSS was applied. In the DSS model these 5 days are followed by 5 days without DSS. During this time wound healing takes place. The authors should also study this time period since it is not unlikely that lack of MMP13 might have an effect during the recovery phase.*'

The chemical DSS model is one of inflammatory bowel disease (IBD) models that is of great value for a better understanding of acute inflammatory disease processes in IBD. During the recovery phase, also other processes such as wound healing play an important role in the obtained clinical score. We studied the mice until day 10 and, as expected based on previous publications that implicate a crucial role for MMP13 in wound healing, the protection of the MMP13$^{-/-}$ mice is less pronounced during the recovery phase (see rebuttal Fig. 10). We included this in the revised manuscript.

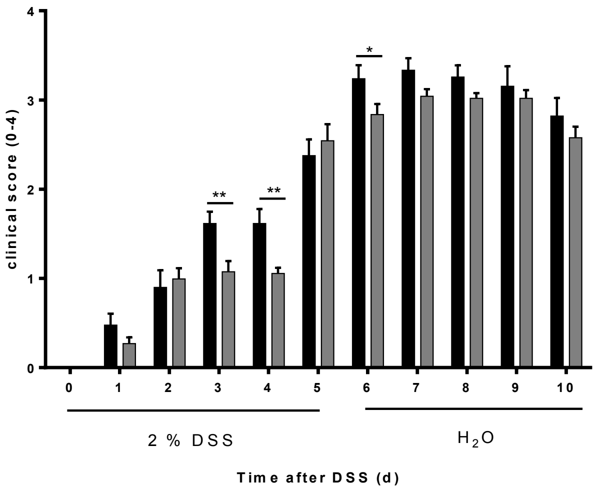


Fig. 10: Clinical score of DSS treated MMP13$^{+/+}$ (black) and MMP13$^{-/-}$ (grey) mice.

(9) '*The authors mention the 'uncleavable TNFa', which has been used in the study described by Mueller et al. (1999). In this TNFa cleavage by ADAM17 has been abolished by introducing 3 point mutations close to the cleavage site. It would be interesting to know whether this mutant TNFa is still cleaved by MMP13.*'

This is an interesting question. However, it is not easy to address it. For the *in vitro* cleavage assay and the determination of the MMP13 cleavage sites, we made use of human recombinant proTNF. In contrast, the mice described by Mueller et al. (1999) contain mutations in mouse TNF and there is too much sequence difference between mouse and human TNF to predict exactly were the cleavage will occur in mouse TNF.

(10) '*On p13 of the manuscript the authors write: 'The importance of the intestinal bacteria was further strengthened by the observation that sterile MMP13$^{+/+}$ mice were more resistant than MMP13$^{-/-}$ mice to LPS-induced shock. This experiment is not shown in the manuscript. In Fig. 5E, only wt mice are shown.*'

We apologize for this typo in the original manuscript. Indeed, we showed in this experiment that sterile MMP13$^{+/+}$ mice are more resistant to LPS-induced shock compared to non-sterile MMP13$^{+/+}$ mice. We have corrected this mistake in the revised manuscript.

(11) '*For inflammatory bowel disease it has been shown in many studies that activation of the EGF receptor pathway and activation of the STAT3 pathway is important for wound healing upon DSS application. This should be discussed in view of the experiments presented in the current study.*'

We discussed this in the 'Discussion section' of the revised manuscript.

*Minor Comments:*

(1) '*The study by Le Gall et al (2009) should be taken into account for the discussion of cleavage of one substrate by several proteases. This study demonstrates that not only ADAM17 but also ADAM10 (in the absence of ADAM17) can efficiently cleave TNFa. This might also apply to MMP13.*'

We now included this study in the Discussion of the manuscript. Additionally, we measured Adam10 and Adam17 levels and did not find any differences between MMP13$^{+/+}$ and MMP13$^{-/-}$ mice (rebuttal Fig. 9). These data were included in the revised manuscript as supplemental data.

2. '*In Fig. 4D and Fig. 6D the authors define the activity of TNFa in U/mg tissue. They should also give the TNFa levels in pg/ml as in Fig. 1H.*'

TNF levels were either determined by bioplex or bioassay. We used bioplex analysis because we wanted to analyse 23 different cyto- and chemokines all at once in serum and the readout of this analysis is in pg/ml. Moreover, soluble TNF that is present in the analysed serum, is always active. In contrast, in our ileum lysates we only wanted to detect activated, cleaved TNF. Therefore, we switched to bioassay and this results in units/mg tissue. We clarified this in the Materials and methods section of the revised manuscript.

(3) '*The different amounts of LPS used in different experiments should be commented and explained (p15).*'

This is now described more clearly in the materials and methods section of the revised manuscript.

(4) '*Some references are not correctly formatted.*'

The references were carefully checked and changed if needed.

Response to referee #3:

'*The techniques used in this study are of very good quality. Similarly, the statistical analysis was done correctly.'*

*'The novelty of this study is medium because it had previously shown that:*

*-LPS increases paracellular permeability and bacterial translocation participating to sepsis.*

*-LPS induces the secretion of TNF*

*-TNF increases the paracellular permeability by caveolin mediating endocytosis and/or induction of MLCK expression and activity.*

*The novelty is the role of MMP13 in the cleavage of TNF.'*

*'The medical impact of this discovery is currently considered average but could be higher if inhibitors of MMP13 were available.'*

*'The models used in this study are adequate.*'

'*The group of Claude Libert provides an interesting manuscript showing that an induction of MMP13 expression and activity in response to LPS injection alters epithelial barrier function causing mice death and colitis severity induced by DSS infusion. Thus, MMP13-/- mice display a strong protection toward sepsis induced by LPS. This protection is linked to an absence or a reduction of the LPS ability to trigger goblet cell depletion, ER stress, permeability, and tight junction destabilization through a mechanism depending of the MMP13. The authors also reported that MMP13 is able to cleave pro-TNF into bioactive TNF supporting that MMP13 is an alternative pathway (in contrast with the TNF converting enzyme TACE) to mature TNFa. Finally, similar mechanism was responsible for the observed protection of the MMP13-/- mice in a mouse model of DSS-induced colitis.*'

'*In general the paper is well written and this is a presentation of well-performed experiments that are carefully interpreted. Nevertheless some controls are missing and the demonstration of the mechanism suffers of some weakness. Finally, there is no molecular data inside cell evidencing how the LPS induced MMP13 expression and activity. All of these criticisms are detailed below.*'

*Major comments:*

(1) '*To explain the mechanism by which LPS increases the paracellular permeability, the authors have evidenced that TNFa induces caveolin-dependent endocytosis of some of tight junction proteins. Nevertheless, the experience of cell fractionation is not enough to support such a conclusion. The authors should demonstrate by using inhibitors of the different endocytosis pathways that caveolin is responsible for the endocytosis of tight junction protein.*'

This is an interesting question and was addressed by Marchiando et al. (2010). In this paper, the authors nicely show that TNF, after binding with TNFR1, induces caveolin-dependent endocytosis of tight junction proteins, which was proven by studies with eGFP-occludin mice and endocytosis inhibitors. This paper is discussed in the 'Discussion section' of the manuscript.

(2) '*The authors have evidenced that LPS increases the paracellular permeability by a mechanism involving caveolin endocytosis. However, a robust literature clearly evidenced that LPS alters paracellular permeability by a mechanism involving the myosin light chain kinase (MLCK) and not the endocytosis. How the authors explain this discrepancy? and it would be nice whether the role of the MLCK might be invalidated or validated. For that a treatment with MLCK inhibitors (ML-7, ML-9) should be performed.*'

This issue was also addressed in the paper of Marchiando et al. (2010). They claim that both processes, namely the TNF-dependent MLCK phosphorylation and endocytosis are closely linked and should not be considered as two completely different mechanisms.

(3) '*To reinforce the role of the TNFa in the death induced by LPS, a control is lacking. Although TNFa treatment of $MMP13^{-/-}$ mice suppresses the protection toward the death induced by LPS, the authors should absolutely evidence that $MMP13^{+/+}$ mice treated with anti-TNF antibodies present a better survival in response to LPS.*'

We thank the reviewer for this suggestion, which was also made by referee #2. As shown in Fig. 8A, TNF neutralization protects mice from LPS-induced lethality. In agreement with this, the same treatment was associated with a reduction in LPS-induced intestinal permeability (Fig. 8B).

Moreover, mucine-2 staining of ileal sections revealed more mucus containing Goblet cells anti-TNF treated mice compared to PBS treated mice after LPS challenge (Fig. 8C and 8D). These results clearly show strong similarity in the protective mechanism of anti-TNF treatment and absence of MMP13. These data are included in the revised manuscript as Fig. 5F-5I.

(4) '*The authors evidenced that TNFa secreted in response to LPS provokes mice death. However, we did not know which type of TNF receptors is involved? Is it the receptor type1 or 2?*'

In general, it is believed that mainly TNFR1 plays a role in TNF-induced toxicity. To address this, we injected TNFR1$^{-/-}$ mice with LPS and, as expected, these mice were significantly protected against LPS-induced lethality (rebuttal Fig. 11). This was included in the revised manuscript as supplemental material.

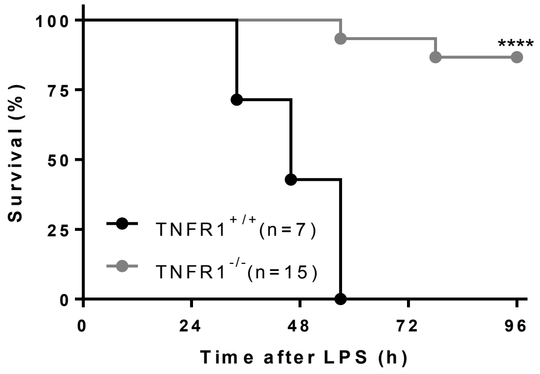


Fig. 11: Survival curve of TNFR1$^{+/+}$ and TNFR1$^{-/-}$ mice injected with LPS.

(5) '*The authors evidenced that TNF induces mucus secretion, goblet cell depletion as well as ER stress. In addition, the authors have shown that mucus depletion in the MMP13$^{-/-}$ mice induced by pilocarpine present a similar rate of mortality than observed in the MMP13$^{+/+}$ mice treated with LPS. What is the mechanism by which TNF induces the depletion of mucus and goblet cells? Is it the induction of ER, which triggers the depletion of mucus and goblet cells? If it is the case the authors should show that the inhibition of ER by tudca treatment ameliorates the survival rate. In addition, the authors have evidenced no apoptosis in response to LPS. What is the mechanism by which LPS decreases the number of Goblet cells? Finally, as BIP is an essential chaperon protein, it is very surprise that no staining of BIP was observed in MMP13$^{-/-}$ after LPS treatment. To reinforce the induction of ER, the protein expression of CHOP should be tested. Moreover, as CHOP is pro-apoptotic protein, this will allow the authors to exclude an involvement of apoptose.*'

Indeed, as suggested by the referee, we believe that TNF induces ER stress in the Goblet cells, which results in a reduction in protective mucus layer, followed by increased bacterial interaction with the epithelial cell layer. In rebuttal Fig. 12 [deleted] we show the TNF-dependent induction of ER stress markers in goblet cells *in vitro*. Although we believe that these questions are important, we prefer not to include these data in the current manuscript. Moreover, these data are part of a recently finished and submitted story about the exact mechanism of TNF-induced intestinal damage of our research team.

Fig. 12: BiP and IRE1 gene expression analysis after LPS stimulation of goblet cells *in vitro*.

(6) 'The authors claim that the death of mice induced by LPS is linked with an increase of paracellular permeability and the depletion of both mucus and goblet cells. What is the most important parameter involved in the mice death? Is it the increase of paracellular permeability or the depletion of mucus and goblet cells? To test these hypotheses, it would be nice to treat the mice with

inhibitors of the caveolin-endocytosis to block the increase of paracellular permeability and to monitor the mice death. In addition, the use of Math1flox/flox mice depleted for goblet cells could allow testing the involvement of goblet cell in the mice survival in response to LPS.'

Although the use of Math1flox/flox mice in the endotoxemia model would be informative, these mice are also devoid of other secretory cells, such as Paneth cells. Paneth cells are responsible for the production of anti-microbial peptides and therefore severely affect the microbial composition. Consequently, the LPS response of these mice would not exclusively be dependent on the absence of goblet cells and therefore extremely difficult to interpret.

We do believe that, based on the fact that mucus depletion can sensitize MMP13$^{-/-}$ mice (rebuttal Fig. 7), the absence of mucus is the most important parameter. Absence of mucus results in increased interaction of commensal bacteria with the epithelial cell layer, increased local inflammation and increased paracellular leakage. The latter results in leakage of bacteria and/or bacterial components into the bloodstream, which results in uncontrolled systemic inflammation and eventually death.

(7) '*To reinforce the role of MMP13 in the response to LPS, the authors should test the expression and the activity of the TACE following LPS injection. In addition the use of KO mice for TACE expression would be more significant*.'

We measured TACE expression in MMP13$^{+/+}$ and MMP13$^{-/-}$ mice; both in unstimulated and stimulated conditions and this did not reveal any differences (rebuttal Fig. 3). The use of TACE$^{-/-}$ mice would indeed be informative. However, full knockouts mice are not viable and conditional knockout mice were not available for our studies.

(8) '*It is unfortunate that we have no data concerning the molecular mechanism by which LPS increase expression and activity of MMP13.*'

We detected MMP13 up-regulation after LPS stimulation in several organs, but we did not have a closer look at the molecular mechanism that regulates this expression. According to literature, several transcription factor-binding sites are present (rebuttal Fig. 13). MMP13 is e.g. transcriptionally activated by IL-1, via the p38 pathway interacting at the MMP-13 promoter through the tissue-specific transcription factor Runx-2 and the ubiquitous AP-1 transcription factor (Mengshol at al., 2011). CCAAT/enhancer binding protein beta (C/EBPbeta) was also shown to directly bind to the promotor region of MMP13 and activate its expression in inflammatory arthritis (Hayashida et al., 2009). So, based on literature, MMP13 mRNA expression is up-regulated after several inflammatory stimuli.

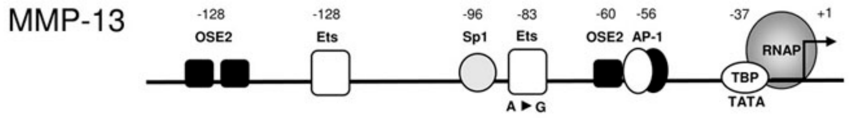


Fig. 13: Schematic overview of the MMP13 promotor as described in Rowan & Young (2007).

(9) '*The authors will show that antibiotic treatment is effective in inducing decontamination or at least a significant reduction of the bacterial load to validate their results.*'

We described in the materials and methods section how we checked the effectiveness of the antibiotic treatment.

(10) '*To reinforce the impact of TNF in the regulation of Muc2 expression, the quantification of protein level is absolutely required. At least, in HT29MTX model, only the mRNA level has been performed.*'

As shown in the original manuscript, we analysed mucin-2 mRNA expression after incubation of HT29-MTX cells with TNF, which revealed huge up-regulation. Due to the size and glycosylation status of mucins, mucin protein is not that easy to detect by western blot. However, the effect of TNF on mucin-2 protein secretion was already described in literature in the paper of Smirnova et al. (2001). Here, they show that incubation of HT29-MTX cells with TNF induces mucin secretion within the first 24 h, with peak response at 7 h after induction. This is now included in the revised manuscript.

*Minor comments:*

(1) '*The data concerning the apoptosis (TUNEL) are important and it should be including in the supplementary file.*'

These data were included in the revised manuscript as supplemental material.

(2) '*Muc2 immunostaining is an important data and it should be including in the supplementary file.*'

These immunostainings were included in the supplementary material of the revised manuscript.

(3) '*Although the authors have performed immunostaining of BIP (qualitative approach), a quantitative approach such as western blot is required.*'

We believe that the BiP immunostainings are more informative than western blot analysis. The confocal images were all taken with exactly the same laser and detector settings, so the analysis can be considered semi-quantitative. We did try western blot analysis with the same antibody, however, this was not successful.

(4) '*The picture of the western blot for ZO-1, Occludin and Claudin 1 should be including the Fig. 3.*'

Representative western blot images were included in Fig. 3 of the revised manuscript.

(5) '*In the Fig. 7, the authors present a synthetic schema of the mechanism by which LPS alters the homeostasis of the gut mucosa. However, the bacteria did not translocate across gut mucosa through tight junction, but across the cells (transcellular pathway). Moreover, the maximum aperture of the tight junctions is 120A. Please can you delete or move the arrow number 4.*'

Fig. 7 was partially adapted according to the suggestions of the referee. Once tight junctions are dysfunctional, we do believe that bacteria can translocate trough paracellular transport.

2nd Editorial Decision 08 April 2013

Thank you for the submission of your revised manuscript to EMBO Molecular Medicine. We have now received the enclosed reports from the referees that were asked to re-assess it. As you will see, reviewers #1 and #2 are now supportive while referee #3 remains concerned about a certain number of issues. However, as we discussed in our previous letter, the revised manuscript focused on addressing satisfactorily the concerns from referees #1 and #2, and as such I am pleased to inform you that we will be able to accept your manuscript pending the following final amendments:

- we realized that you have provided figures within your rebuttal letter. As you know, EMBO publishes the referees' reports and responses from the authors as part of its Transparent policy. Would you agree to have these figures published within this Review Process File or would you rather remove them?
-We now encourage the publication of source data, particularly for electrophoretic gels and blots, with the aim of making primary data more accessible and transparent to the reader. Would you be willing to provide a PDF file per figure that contains the original, uncropped and unprocessed scans of all or key gels used in figures 3 and 4? The PDF files should be labeled with the appropriate figure/panel number, and should have molecular weight markers; further annotation may be useful but is not essential. The PDF files will be published online with the article as supplementary "Source Data" files. If you have any questions regarding this just contact me.

I look forward to reading a new revised version of your manuscript as soon as possible.

***** Reviewer's comments *****

Referee #1 (General Remarks):

This is an interesting manuscript that now provides extensive evidence to suggest that MMP13 could be considered as a potential therapeutic target for the treatment of septic conditions.

In the revised version of the manuscript, the authors have put forth a large effort to address and resolve the concerns raised by the reviewers, and the paper is significantly improved. The new experiments with MMP13 inhibitor in primary macrophages and in ileum tissue explants significantly strengthen the link between MMP13 and the cleavage of TNF, which was further validated as key initiator of the intestinal inflammation and epithelial barrier disruption affected by MMP13 deficiency. With new further data from the expression of other MMPs to result validation using alternative model, the revised manuscript now convinces this reviewer.

Referee #2 (Comments on Novelty/Model System):

This is a very thoroughly revised version of the manuscript, which should be acceptable now. This might even be a paper, which should somehow be highlighted with a comment (perspective article). It is very surprising that mice without MMP13 but with ADAM17 have a very similar response to septic shock as mice without ADAM17 but with MMP13. There is communication between proteases that is not understood at the molecular level so far.

Referee #2 (General Remarks):

The manuscript describes a study with MMP13-/- mice. These mice are protected from a bolus injection of LPS, which proved to be lethal in wt animals. The authors attributed the protection to reduced goblet cell depletion, ER stress, enhanced intestinal permeability and tight junction destabilization in the gut. The authors found in addition that MMP13 was able to cleave TNF in vitro at sites adjacent to the natural TNF cleavage site used by ADAM17. Furthermore, the authors found that MMP13-/- mice are less affected during the acute phase of DSS colitis as compared to wt

© EMBO 

mice. The authors conclude that MMP13 is an additional TNF converting enzyme and hypothesize that lack of TNF cleavage is the reason why MMP13-/- mice are protected from death following LPS injection.

This is an interesting study, which indicates that MMP13 could be a therapeutic target for the treatment of septic conditions and inflammatory bowel disease.

All points raised by this reviewer have been satisfactorily addressed.

Referee #3 (Comments on Novelty/Model System):

The techniques and statistical analysis used in this study are of very good quality.

The novelty of this study is medium because it had previously shown that:
-LPS increases paracellular permeability and bacterial translocation participating to sepsis.
-LPS induces the secretion of TNF
-TNF increases the paracellular permeability by caveolin mediating endocytosis and/or induction of MLCK expression and activity.

The novelty is the role of MMP13 in the cleavage of TNF.

3. The medical impact of this discovery is currently considered average but could be higher if inhibitors of MMP13 were available.

4. The models used in this study are adequate.

Referee #3 (General Remarks):

The revised version of the manuscript presented a numerous novel data greatly improving the strength of the paper. Thus, the authors have performed additional experiments reinforcing the main role of MMP13 in the maturation of TNF. In addition, a new model of sepsis shock has been developed and included in the revised version of the manuscript. Although numerous criticisms have been correctly addressed some major concerns have not been addressed:

1. The mechanism by which LPS alters the paracellular permeability is poorly investigated and very speculative. Thus, the authors have evidenced that TNF induces caveolin-dependent endocytosis of some of tight junction proteins. Nevertheless, the experience of cell fractionation is not enough to support such a conclusion. The authors should demonstrate by using inhibitors of the different endocytosis pathways that caveolin is responsible for the endocytosis of tight junction protein. For inhibitors see the paper of Marchiando et al. JCB 2010.

2. The authors evidenced that TNF induces mucus secretion, goblet cell depletion as well as ER stress. What is the mechanism by which TNF induces the depletion of mucus and goblet cells? Is it the induction of ER which triggers the depletion of mucus and goblet cells? Again this main question is not addressed and the answer is very speculative.

3. The authors claim that the death of mice induced by LPS is linked with an increase of paracellular permeability and the depletion of both mucus and goblet cells. What is the most important parameter involved in the mice death? Is it the increase of paracellular permeability or the depletion of mucus and goblet cells? Again the response is speculative

Finally, although there is no data in this paper evidencing that bacteria translocates by paracellular pathway, the authors present again a synthetic schema of the mechanism by which LPS induced bacterial translocation across tight junction. To my knowledge there is NO PAPER evidencing that bacteria translocate across paracellular pathway. In contrast, since the authors poorly evidenced or suggested that thigh junction is altered by the induction of endocytosis event, why the authors do not

hypothesized that bacteria could be included in these endocytosis vacuoles and translocates via transcellular route. Moreover a rich literature evidences that bacteria translotes by TRANSCELLULAR PATHWAY. Please can you delete or move the arrow number 4.
