# Supplementary material for: Matrix metalloproteinase 13 modulates intestinal epithelial barrier integrity in inflammatory diseases by activating TNF
Source: EMBO Mol Med. 2013 May 30;5(7):932–48. doi: 10.1002/emmm.201202100 (PMC3721470; doi:10.1002/emmm.201202100)

Source data – Vandenbroucke et al. 2013 – Matrix metalloproteinase 13 modulates intestinal epithelial barrier integrity in inflammatory diseases by activating TNF

Precision Marker (BioRad): 250-150-100-75-50-37-25-20-15-10 kDa

Figure 3A

Proteins: ZO1 green (220 kDa) & actin red (42 kDa)
Samples: marker - 3x 0 h MMP13$^{+/+}$ - 3x 0 h MMP13$^{-/-}$ - 4x 0 h MMP13$^{+/+}$ - 4x 0 h MMP13$^{-/-}$

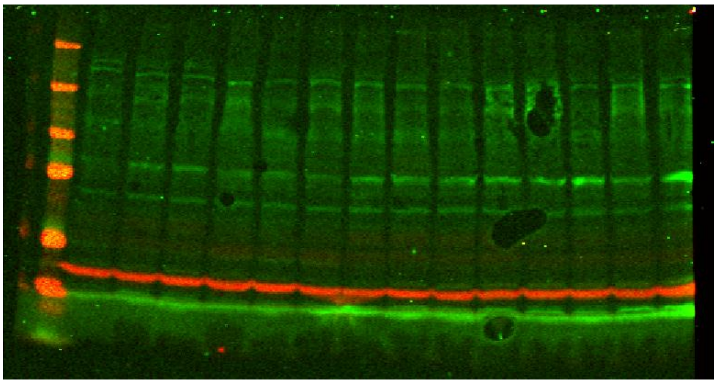

Figure 3B

Proteins: occludin green (65 kDa) & actin red (42 kDa)
Samples: marker - 3x 0 h MMP13$^{+/+}$ - 3x 0 h MMP13$^{-/-}$ - 4x 0 h MMP13$^{+/+}$ - 4x 0 h MMP13$^{-/-}$

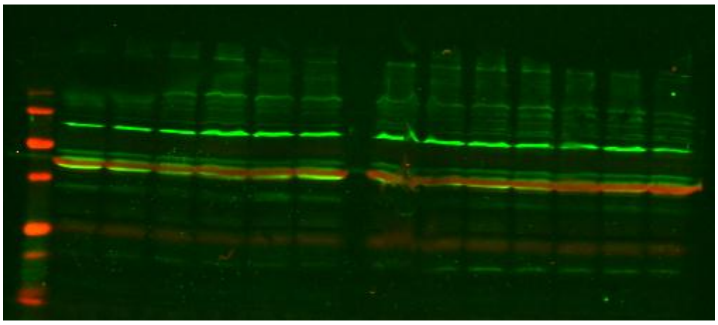

Figure 3C

Proteins: claudin-1 green (23 kDa) & actin red (42 kDa)
Samples: marker - 3x 0 h MMP13$^{+/+}$ - 3x 0 h MMP13$^{-/-}$ - 4x 0 h MMP13$^{+/+}$ - 4x 0 h MMP13$^{-/-}$

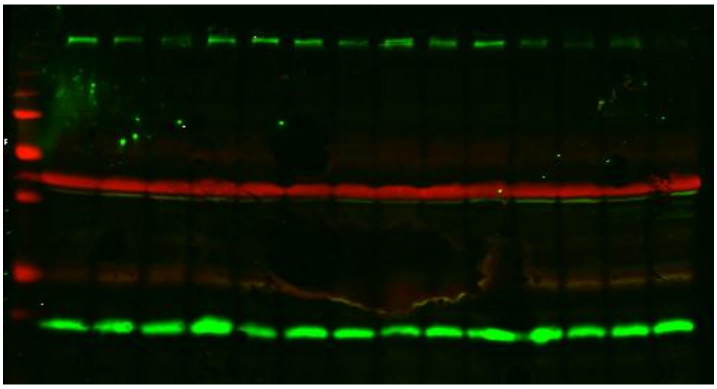

Figure 3G

Protein: caveolin-1 green (23 kDa)
Samples: marker - fractions 2 till 10

MMP13$^{+/+}$ with LPS

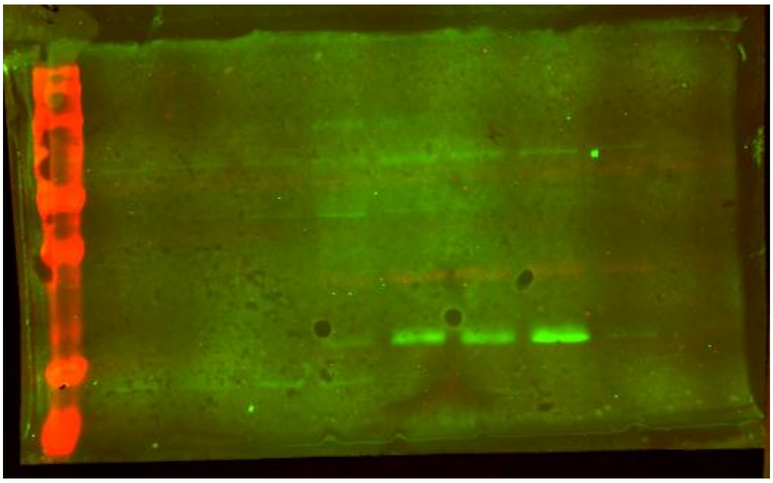

MMP13$^{-/-}$ with LPS

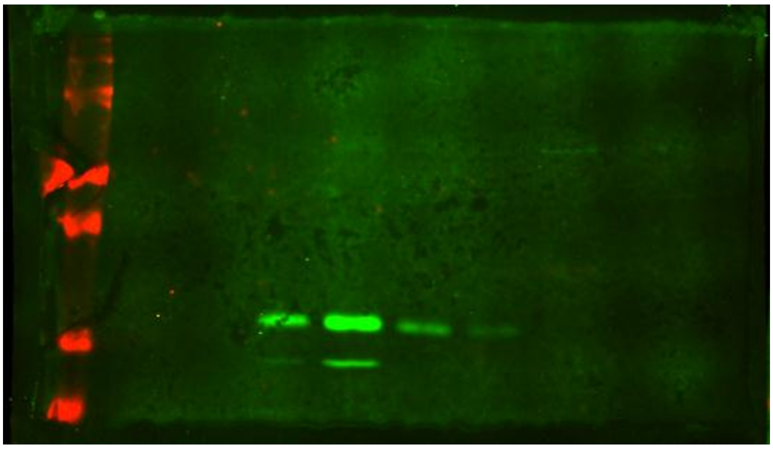

MMP13$^{+/+}$ without LPS

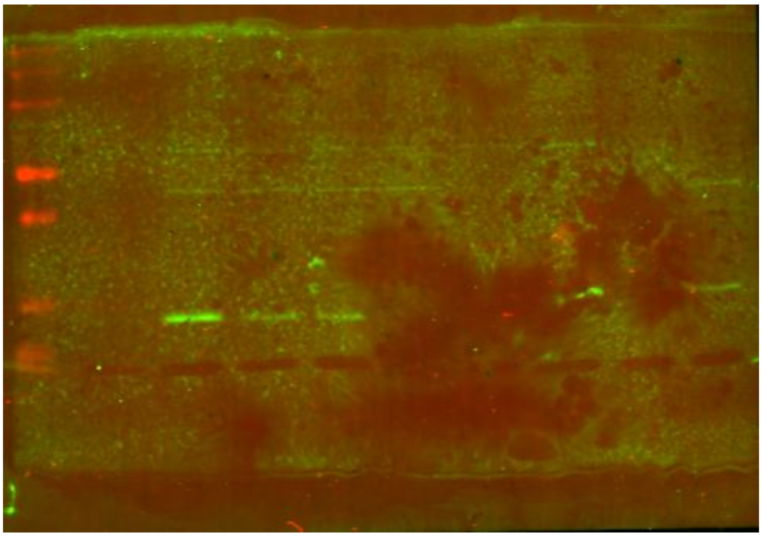

MMP13$^{-/-}$ without LPS

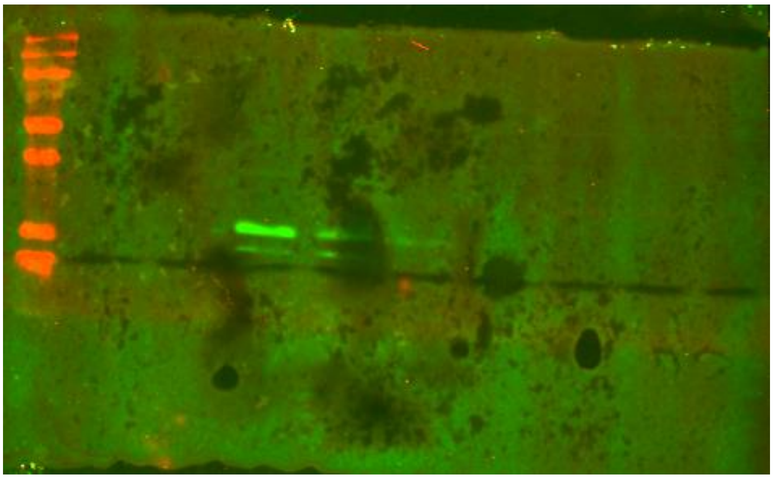

Figure 4A

proTNF – silver staining
Samples: marker – 0 min – 2 min – 5 min – 10 min – 20 min – 30 min

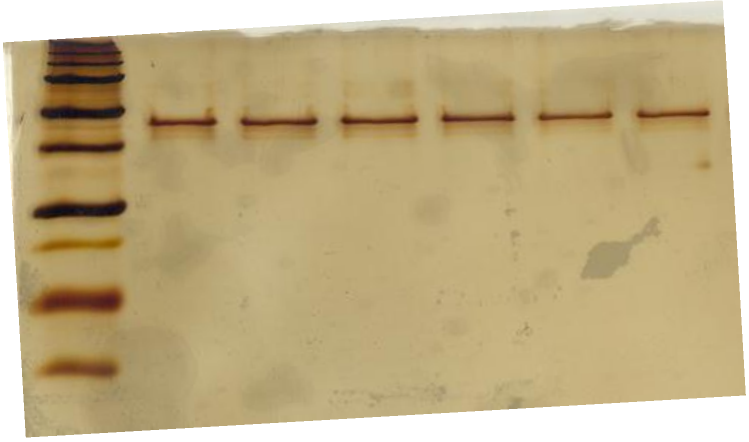

MMP13 – silver staining
Samples: 0 min – marker – 2 min – 5 min – 10 min – 20 min – 30 min

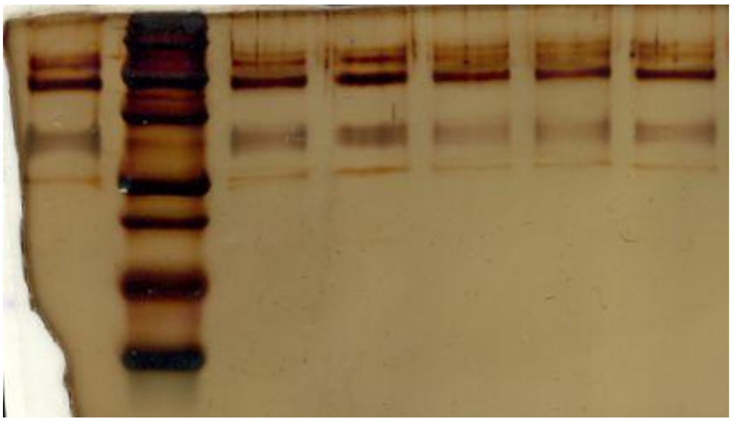

TNF – western blot

Protein: proTNF (42 kDa) and mature TNF (11-17 kDa) green

Samples: 0 min – marker – 2 min – 5 min – 10 min – 20 min – 30 min

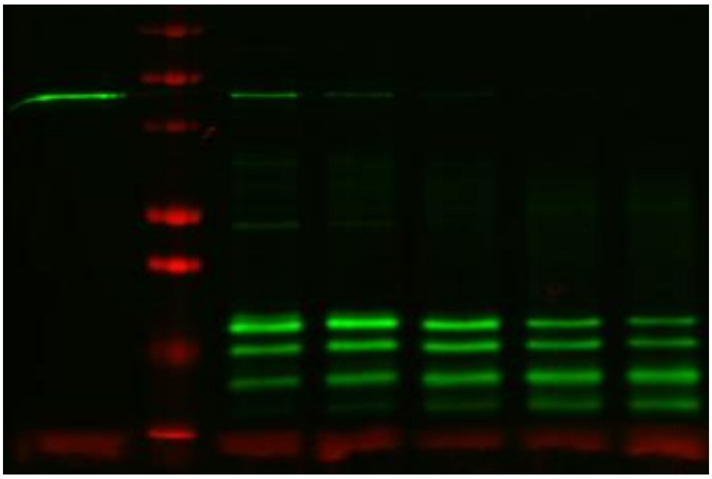

Figure 4B

Protein: proTNF (42 kDa) and mature TNF (11-17 kDa) green

Samples: marker – proTNF – proTNF/TACE – proTNF/MMP13

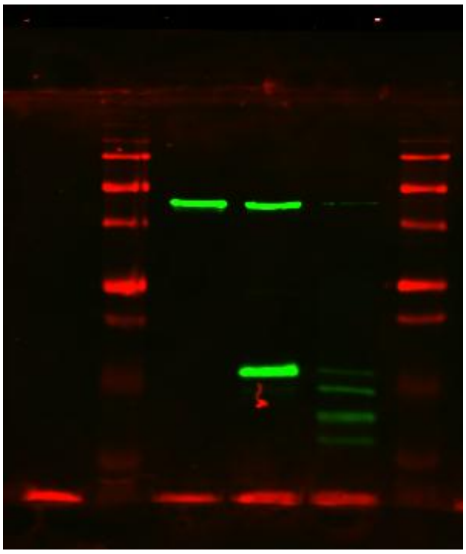

Supplement: Supplementary file 2 [file emmm0005-0932-SD2.pdf]
