# Supplementary material for: Matrix metalloproteinase 13 modulates intestinal epithelial barrier integrity in inflammatory diseases by activating TNF
Source: EMBO Mol Med. 2013 May 30;5(7):932–48. doi: 10.1002/emmm.201202100 (PMC3721470; doi:10.1002/emmm.201202100)
Supplement: Supplementary file 3 [file emmm0005-0932-SD3.pdf]

Vandenbroucke et al. 2013 - Matrix metalloproteinase 13 modulates intestinal epithelial barrier integrity in inflammatory diseases by activating TNF

Table of content:

Supplemental Figure S1.

Supplemental Figure S2.

Supplemental Figure S3.

Supplemental Figure S4.

Supplemental Figure S5.

Supplemental Figure S6.

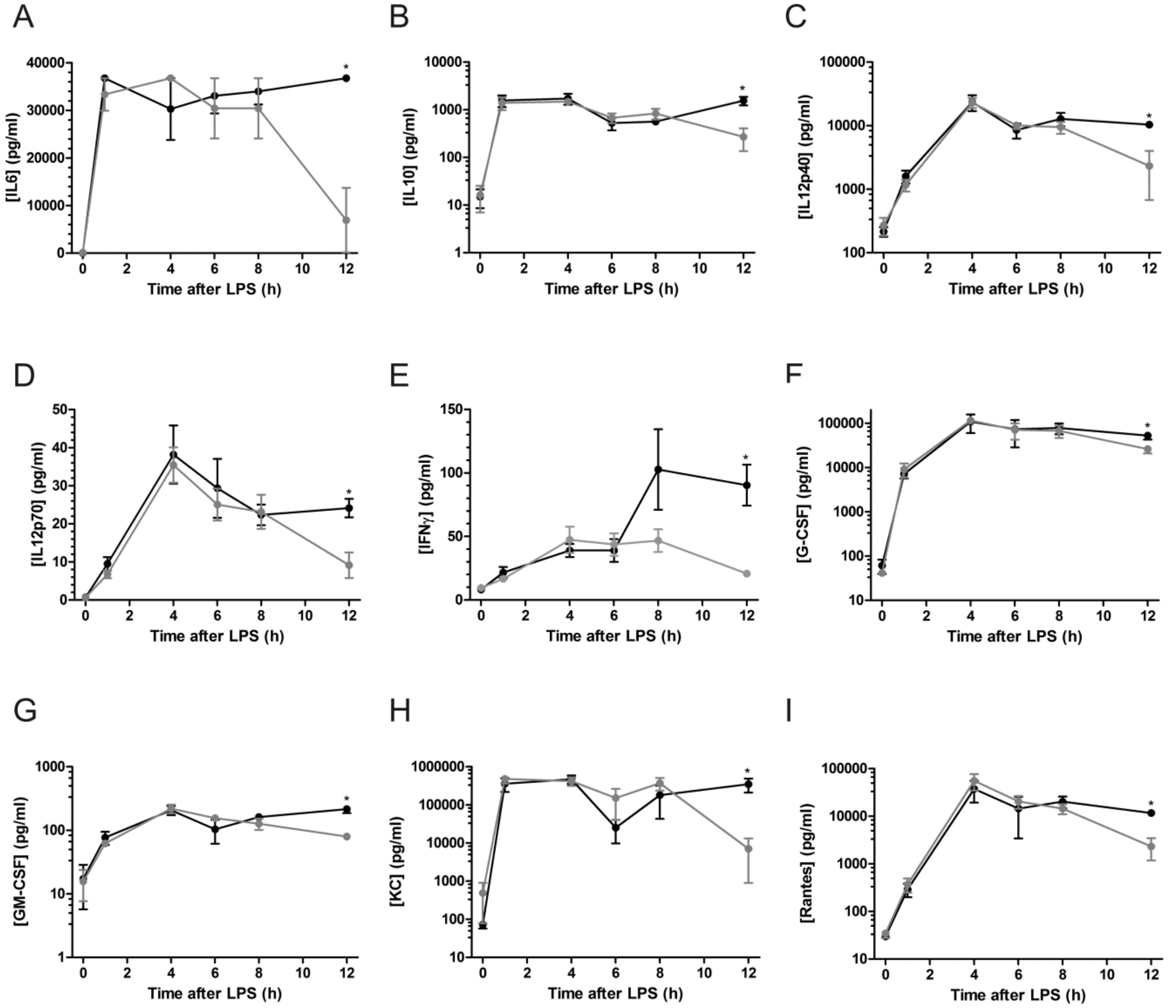

Supplemental Figure S1. MMP13 deficiency protects mice from LPS-induced systemic inflammation. **(A-I)** Serum cytokine and chemokine levels after injection of LPS in $MMP13^{+/+}$ (black) and $MMP13^{-/-}$ (grey) mice: IL6 (A), IL10 (B), IL12p40 (C), IL12p70 (D), IFNγ (E), G-CSF (F), GM-CSF (G), KC (H), and Rantes (I).

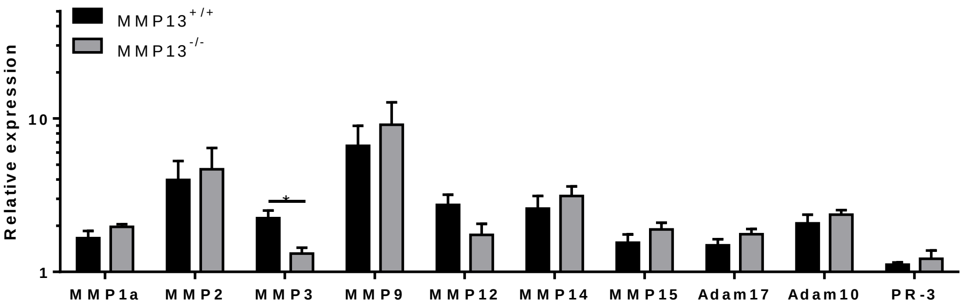

Supplemental Figure S2. MMP expression in unstimulated MMP13$^{+/+}$ (black) and MMP13$^{-/-}$ (grey) mice (n=4-5).

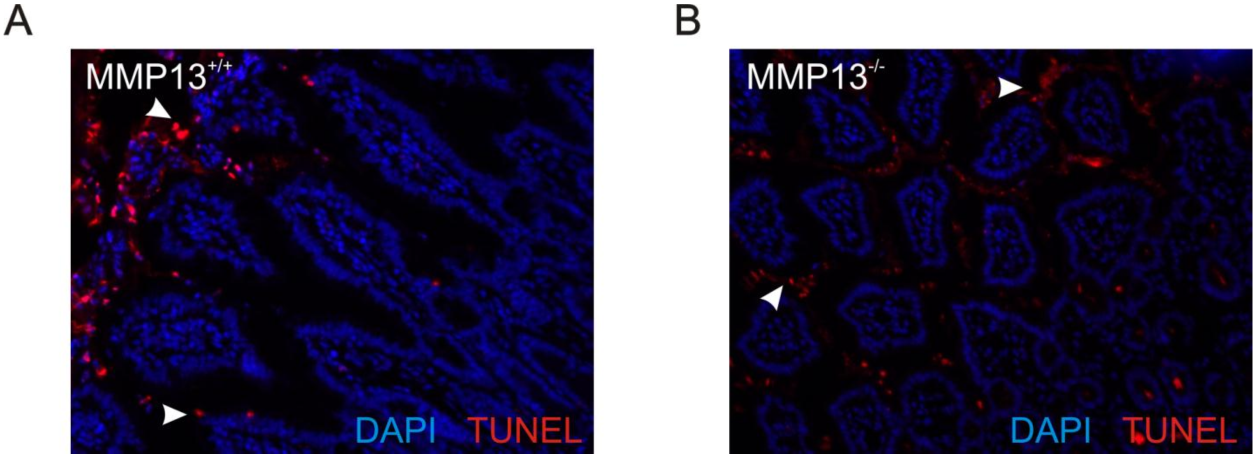

Supplemental Figure S3. Fluorescent images of TUNEL staining (red) in (A) MMP13$^{+/+}$ and (B) MMP13$^{-/-}$ mice, eight hours after LPS stimulation. Nuclei are stained by DAPI (blue).

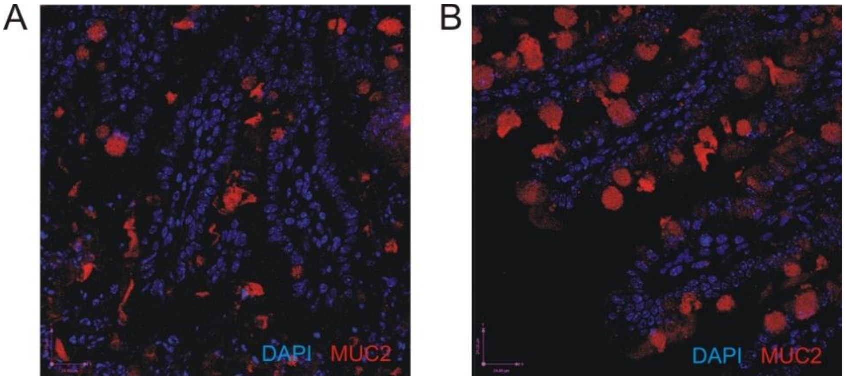

Supplemental Figure S4. Mucin-2 (red) immunostaining of ileum sections of (A) MMP13$^{+/+}$ and (B) MMP13$^{-/-}$ mice 24 h after LPS injection. Nuclei are stained by DAPI (blue).

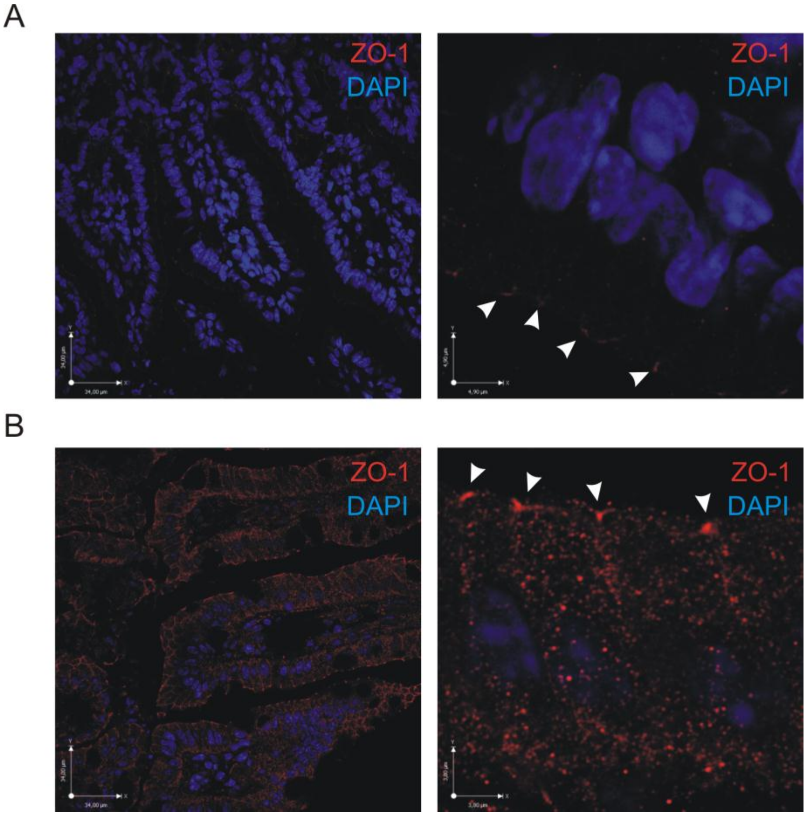

Supplemental Figure S5. (A-B) Confocal images of ZO-1 staining in the ileum of MMP13$^{+/+}$ (A) and MMP13$^{-/-}$ (B) mice 8 h after LPS injection. Nuclei are stained by DAPI (blue). The arrows indicate the position of the tight junctions.

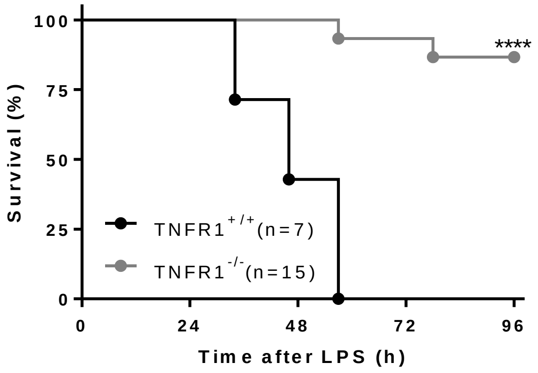

Supplemental Figure S6. Survival curve of TNFR1$^{+/+}$ (black; n=7) and TNFR1$^{-/-}$ (grey; n=15) mice injected with LPS.
